# NTRK kinase domain mutations in cancer variably impact sensitivity to type I and type II inhibitors

**DOI:** 10.1038/s42003-020-01508-w

**Published:** 2020-12-16

**Authors:** Romel Somwar, Nicolle E. Hofmann, Bryan Smith, Igor Odintsov, Morana Vojnic, Irina Linkov, Ashley Tam, Inna Khodos, Marissa S. Mattar, Elisa de Stanchina, Daniel Flynn, Marc Ladanyi, Alexander Drilon, Ujwal Shinde, Monika A. Davare

**Affiliations:** 1 Department of Pathology, Memorial Sloan Kettering Cancer Center, New York, NY USA; 2 Human Oncology and Pathogenesis Program, Memorial Sloan Kettering Cancer Center, New York, NY USA; 3 Department of Pediatrics, Oregon Health & Science University, Portland, OR USA; 4 Deciphera Pharmaceuticals, 200 Smith Street, Waltham, MA USA; 5 Antitumor Assessment Core Facility, Department of Pharmacology, Memorial Sloan Kettering Cancer Center, New York, NY USA; 6 Thoracic Oncology Service, Division of Solid Tumor Oncology, Department of Medicine, Memorial Sloan Kettering Cancer Center, New York, NY USA; 7 Department of Chemical Physiology and Biochemistry, Oregon Health & Science University, Portland, OR USA

**Keywords:** Molecular modelling, Oncogenes, Targeted therapies

## Abstract

Tyrosine kinase domains dynamically fluctuate between two main structural forms that are referred to as type I (DFG-in) or type II (DFG-out) conformations. Comprehensive data comparing type I and type II inhibitors are currently lacking for NTRK fusion-driven cancers. Here we used a type II NTRK inhibitor, altiratinib, as a model compound to investigate its inhibitory potential for larotrectinib (type I inhibitor)-resistant mutations in NTRK. Our study shows that a subset of larotrectinib-resistant NTRK1 mutations (V573M, F589L and G667C) retains sensitivity to altiratinib, while the NTRK1^V573M^ and xDFG motif NTRK1^G667C^ mutations are highly sensitive to type II inhibitors, including altiratinib, cabozantinib and foretinib. Moreover, molecular modeling suggests that the introduction of a sulfur moiety in the binding pocket, via methionine or cysteine substitutions, specifically renders the mutant kinase hypersensitive to type II inhibitors. Future precision treatment strategies may benefit from selective targeting of these kinase mutants based on our findings.

## Abstract (web summary)

Romel Somwar et al. find that cancer-causing *NTRK* gene fusions resistant to one form of inhibitor therapy can be resistant to other inhibitor types. Using molecular simulations, they show that some *NTRK1* mutations resistant to the type I inhibitor larotrectinib are hypersensitive to the type II inhibitor altiratinib, potentially due to the introduction of a sulfur moiety in the kinase binding pocket.

## Introduction

Constitutive activation of tyrosine or serine/threonine kinases in cancer can occur via somatic mutation, small deletions or insertions, or chromosomal rearrangements that generate gene fusions wherein the kinase domain of a proto-oncogenic kinase is fused in-frame to the 5′ region of a partner gene. Gene fusions involving the tropomyosin receptor kinases that are encoded by the *NTRK* genes are oncogenic drivers in cancers originating from diverse cell lineages in multiple adult and pediatric solid and hematological malignancies^1,2^. Given their robust oncogenic drive and pharmacologic targetability, kinase fusions have garnered substantial attention as therapeutic targets in cancer, including the noteworthy first fusion to be targeted, BCR-ABL, followed by ALK, ROS1, and NTRK fusions^3–11^.

Durable clinical response to kinase inhibitors is frequently curtailed by the development of therapeutic resistance due to the emergence of secondary mutations in the oncogenic kinase (on-target resistance) or bypass signaling from downstream or parallel pathways^12–20^. In the case of on-target resistance mechanisms, the amino acid substitution changes the three-dimensional conformation of the kinase domain substantively enough to reduce or abrogate the binding of the inhibitor. Given the expanding pipeline of inhibitors available for clinical translation, new or repurposed agents can be rapidly implemented if a priori knowledge of their efficacy to circumvent resistant mutation exists.

Kinase domains have structural fluidity and undergo multiple conformational transitions that have a direct impact on inhibitor binding. The two primary conformers of kinase domains are defined based on the position of three sequential amino acids-aspartic acid (D), phenylalanine (F), glycine (G)-(called the DFG motif) residing at the start of the activation loop within the kinase domain. The activation loop is structurally flexible, and its position dictates whether the kinase is in the inactive or DFG-out conformation or the active or DFG-in conformation. Kinase inhibitors have been classically categorized as type I to III^21^, and newer studies with additional structural mode inhibitors, expanding this to type I to V^22^. Type I and type II represent the most common inhibitors in the current research and clinical pipeline. Type I inhibitors, also referred to sometimes as ‘ATP-competitive or (DFG-in)’ inhibitors, occupy the active conformation of the kinase by binding to the ATP pocket^23^. Type II inhibitors, on the other hand, bind the DFG-out conformation of the kinase and occupy a hydrophobic pocket near the ATP-binding site, in addition to binding the ATP-hinge region.

The majority of tyrosine kinase inhibitors (TKI) currently in clinical use are type I inhibitors, thus to date emergent resistant mutations tend to broadly confer functional resistance to inhibitors that bind this active or DFG-in conformation. We have previously profiled small molecules that inhibit the tyrosine kinase activity of ROS1, and established that type II mode inhibitors effectively suppress the catalytic activity of most kinase mutations that dictate resistance to ROS1 type I inhibitors^24^. Notably, our preclinical studies led to repurposing of the type II ROS1 inhibitor cabozantinib for treating an advanced lung cancer patient who developed resistance to the type I inhibitor crizotinib, due to acquisition of a ROS1^D2033N^ mutation^25^.

Somatic point mutations in the kinase domain of NTRK that confer resistance to the type I inhibitors, larotrectinib and entrectinib, have been identified in pre-clinical screens and clinical samples^14,15,17,20^. These mutations commonly reside in the catalytic region of the kinase domain, and include residues in the solvent front of the ATP-binding pocket (solvent-front mutations), the gatekeeper residue that is a conserved hydrophobic amino acid in the active site (gatekeeper mutation), or the amino acid preceding the activation loop DFG motif (xDFG mutation). Mutations at these sites tend to hinder inhibitor binding and/or enhance catalytic function by decreasing the K_M_ for ATP, resulting in increased competition between ATP and inhibitor binding. These mutations in NTRK1 fusion (TPM3-NTRK1) include G595R, F589L, and G667C, with paralogues in NTRK3 (ETV6-NTRK3) including G623R, F617L, and G696A^17,26^. Recent rationally guided drug development has resulted in several second-generation ATP-competitive inhibitors such as repotrectinb^6^, selitrectinib^26^ and taletrectinib (DS-6051b)^27^ that are effective against solvent-front mutations. However, the xDFG mutations continue to pose challenges even for some of these potent second-generation inhibitors. Specifically, NTRK1^G667C^ was shown to be resistant to DS-6051b^27^, and the inhibitory efficacy of selitrectinib for TPM3-NTRK1^G667C^ mutation is reduced forty-fold as compared to wildtype TPM3-NTRK1 fusion^26^.

Mutational perturbation of NTRK kinase domain undoubtedly alters its conformation and impinges on sensitivity to specific inhibitors. Here, we hypothesized that a subset of these mutations may be vulnerable to type II inhibitors, and to test this we have performed in vitro, in vivo, and structural modeling studies using altiratinib, a type II NTRK inhibitor, as compared to the type I inhibitors larotrectinib and entrectinib. These data may inform future structure-guided rational drug design.

## Results

### Effective inhibition of NTRK1 and NTRK3 fusion proteins with type II binding mode inhibitor

**Fig. 1:**
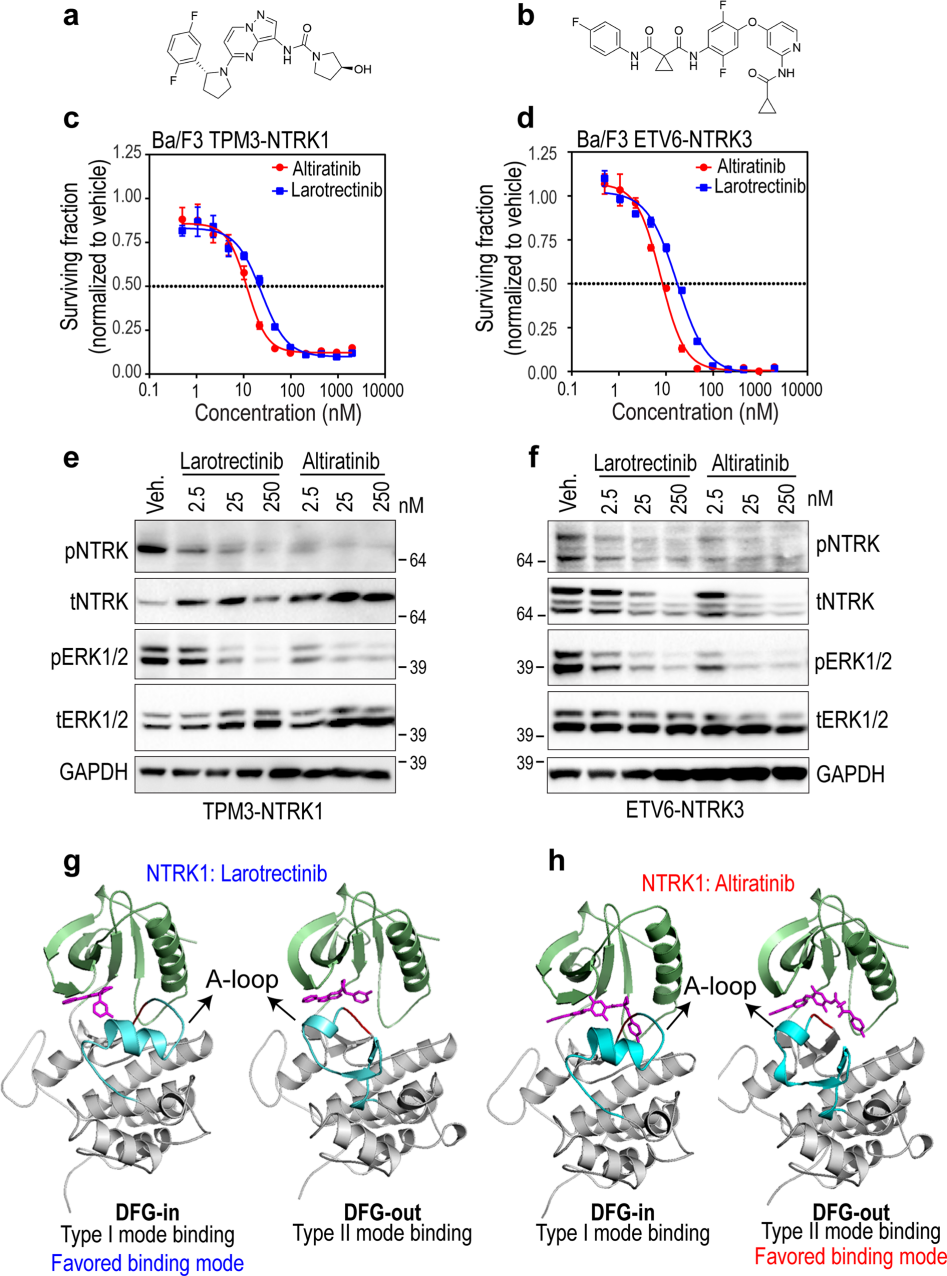
In vitro efficacy and structural basis of binding of larotrectinib and altiratinib for NTRK fusion proteins. Chemical structures of larotrectinib (**a**), and altiratinib (**b**). Dose-response cell viability of onco-addicted Ba/F3 TPM3-NTRK1 (**c**) or ETV6-NTRK3 fusion (**d**) expressing cells after treatment with larotrectinib or altiratinib for 72 h. Representative data are average ± standard error of means (SEM) from three independent replicates. **e**, **f** Immunoblot analysis of TPM3-NTRK1 and ETV6-NTRK3 autophosphorylation (pNTRK), as well as Erk1/2 phosphorylation (pErk1/2), corresponding total NTRK (tNTRK) and Erk1/2 (tErk1/2) levels, and loading control (Gapdh). “Veh.” indicates vehicle (dimethyl sulfoxide (DMSO)) treatment. Data are representative of four independent experiments. Structural homology models of NTRK1 in DFG-in (type I) or DFG-out (type II) conformations bound to larotrectinib (**g**) or altiratinib (**h**); type I and type II are the favored binding modes for larotrectinib and altiratinib, respectively.

The majority of NTRK TKI that are in pre-clinical development or clinical use for the treatment of NTRK fusion-driven malignancies are ATP-competitive or type I inhibitors that preferentially bind to the DFG-in conformation of the kinase domain. Previously described type I NTRK inhibitors include larotrectinib (LOXO-101), entrectinib (RXDX-101), repotrectinib (TPX-0005), and taletrectinib (DS-6051b). In vitro kinase assays using purified recombinant kinase established that altiratinib (DCC-2701) is a potent inhibitor of NTRK1 (IC_50_ = 0.85 ± 0.22 nM), NTRK2 (4.6 ± 0.4 nM) and NTRK3 (0.83 ± 0.39 nM) kinases^28,29^. Notably, altiratinib preferentially occupies the DFG-out conformation of NTRK kinase, and thus is a type II inhibitor^28,29^. Here, we compared the inhibitory potential and structural basis of type I versus type II NTRK TKIs, and utilized larotrectinib as a representative type I inhibitor and altiratinib as type II NTRK TKI. The structures of larotrectinib and altiratinib are shown in Fig. 1a, b. Dose-response cell viability experiments, conducted using well established transformed Ba/F3 cell model system^30^, reveal equivalent potency of larotrectinib and altiratinib with concentrations required to suppress 50% cell growth (IC_50_) for TPM3-NTRK1 driven cells being 26.4 and 11.3 nM, respectively, and for ETV6-NTRK3, 17.2 and 7.3 nM, respectively (Fig. 1c, d). These inhibitors did not suppress proliferation of Ba/F3 parental cells cultured with murine interleukin-3 (mIL-3), a requisite cytokine for cell survival in the absence of any exogenous oncogene; these data confirm the selectivity of these agents for NTRK family kinases (Supplementary Fig. 1). Immunoblotting established on-target suppression of NTRK catalytic activity as measured using a phospho-specific antibody that detects auto-phosphorylation of a highly conserved tyrosine residing in the activation loop, and of key effector pathway proteins, ERK1/2 (Fig. 1e, f). To facilitate structural assessment, we generated larotrectinib- and altiratinib-bound models of the type I (active) and type II (inactive) conformation of the NTRK1 kinase domain using molecular docking approaches (see “Methods”). These docking studies, including the dissociation constants calculated from this analysis, confirmed that altiratinib exhibits preferential binding to NTRK1 in type II (DFG-out, ‘inactive’) conformation, while larotrectinib favors the type I binding mode (DFG-in, ‘active’ conformation) (Fig. 1g, h and Table 1).

**Table 1:** Dissociation constants from molecular docking studies for wildtype and mutant NTRK1 kinases in their DFG-in or DFG-out conformers, as indicated.

Enzyme		Dissociation constant for drug (nM)				
(NTRK1) TrkA	Conformer	Larotrectinib	Entrectinib	Altiratinib	Cabozantinib	Foretinib
WT	Active	100.9	4.6	172.2	301.4	307.5
	Inactive	456.5	11.9	24.1	114.1	54.3
V573M	Active	717.6	32.4	114.5	21.7	24.9
	Inactive	1029.8	58.7	5.75	14.4	14.4
F589L	Active	2708.6	8.5	133.2	237.1	362.4
	Inactive	1116.7	9.3	26.6	200.0	64.6
G667C	Active	5375.1	118.3	18.8	741.6	913.3
	Inactive	4082.3	68.5	11.2	14.1	79.6
G667S	Active	535.9	45.3	377.2	689.2	350.2
	Inactive	2265.4	147.1	81.7	116.3	17.4

Active = DFG-in (type I inhibitor preference).

Inactive = DFG-out (type II inhibitor preference).

The anti-tumor efficacy of larotrectinib has been previously established^31^. Here, we examined the anti-tumor efficacy of altiratinib against NTRK fusion-driven cancer using allograft and xenograft murine model studies. Oral altiratinib (15 mg/kg) treatment of NIH3T3 ETV6-NTRK3 tumors in a subcutaneous murine allograft model abrogated tumor growth, with the expansion of growth upon treatment withdrawal (Supplementary Fig. 2a). The growth of TPM3-NTRK1-driven xenografted tumors generated from KM-12 human colorectal cells was inhibited in a dose-dependent manner with oral altiratinib treatment (Supplementary Fig. 2b). In both models, oral treatment leads to on-target inhibition of NTRK phosphorylation relative to vehicle-treated tumors, in vivo (Supplementary Fig. 2c and d) without measurable toxicity as assessed using body weight measurements (Supplementary Fig. 2e, f). Loss of total ETV6-NTRK3 but not TPM3-NTRK1 is observed both in vivo and in vitro (Fig. 1e, f), and additional experiments described later in the manuscript suggest that sustained catalytic inhibition leads to loss of NTRK3 but not NTRK1 fusion proteins. Taken together these data demonstrate that both the representative type I and type II inhibitors harbor equivalent inhibitory potential against NTRK1 and NTRK3 wildtype kinase domains.

### Comparing potency of type I versus type II inhibitors on solvent front NTRK kinase domain mutations

**Fig. 2:**
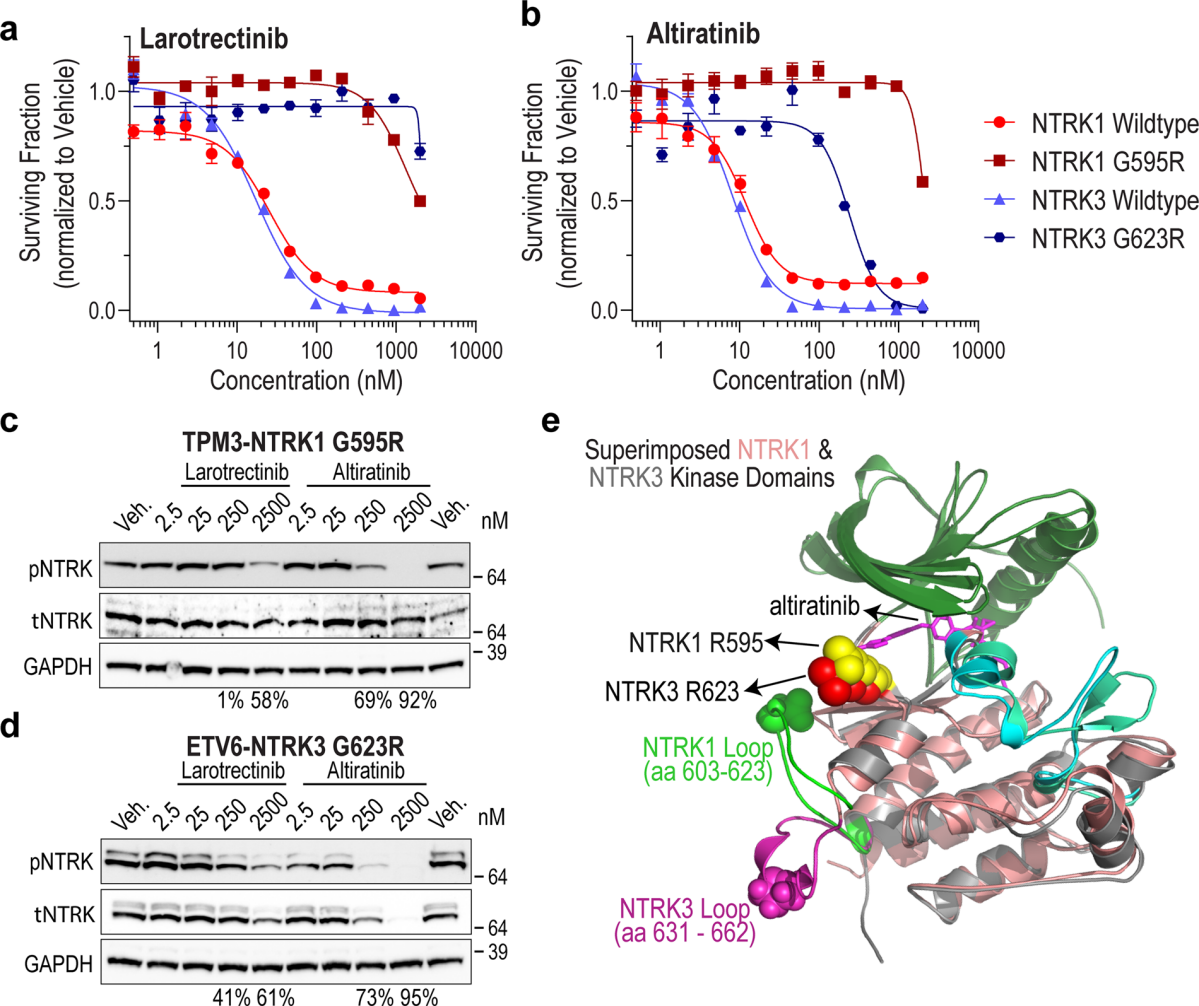
Solvent front mutation TPM3-NTRK1^G595R^ but not ETV6-NTRK3^G623R^ is completely resistant to type I larotrectinib and type II altiratinib inhibitors. Cell viability data for Ba/F3 cells expressing TPM3-NTRK1^G595R^ (**a**) or ETV6-NTRK3^G623R^ (**b**) fusions treated with larotrectinib or altiratinib at the indicated doses after 72 h. Representative data are average ± standard error of means (SEM) from three independent replicates. **c**, **d** Ba/F3 cells expressing TPM3-NTRK1^G595R^ (**c**) or ETV6-NTRK3^G623R^ (**d**) fusions were treated for 2 h with DMSO (Veh: vehicle) or the indicated concentrations (in nanomolar (nM)) of larotrectinib or altiratinib. Immunoblotting to assess TPM3-NTRK1 and ETV6-NTRK3 autophosphorylation (pNTRK), total NTRK (tNTRK), and loading control (Gapdh). Immunoblot data are representative of three independent experiments. The percent inhibition of phosphorylation is shown below the blots for select concentrations. **e** Molecular docking of altiratinib to structural homology models of NTRK1 and NTRK3 solvent front mutants (superimposed alignment of NTRK1 (salmon) and NTRK3 (gray) crystal structures). Green colored loop represents NTRK1 amino acids 603 to 623 and magenta-colored loop represents NTRK3 amino acids 631 to 662.

Despite the efficacy of kinase-fusion targeted inhibitor therapy^5,10,32^, the emergence of drug resistance via the acquisition of novel protein-intrinsic or -extrinsic aberrations limits the durability of tumor response^16,20,33,34^. Gatekeeper or solvent front mutations frequently pose a liability for targeted kinase inhibitors, for example, ALK (ALK^G1202R^) and ROS1 (ROS1^G2032R^) recurrently arise in cancer patients treated with crizotinib, a type I ROS1/ALK/MET targeted TKI^25,35,36^. Mutations in this highly conserved region of the tyrosine kinase domain are frequently observed in the clinical setting for NTRK fusion cases as well^4^. Larotrectinib-resistant solvent front mutations in NTRKs are NTRK1^G595R^ and the paralog in ETV6-NTRK3, NTRK3^G623R^. In the case of ROS1, we previously showed that type II inhibitors effectively block solvent front mutations^24,37^. Here we tested if the type II inhibitor altiratinib would suppress the growth of Ba/F3 TPM3-NTRK1^G595R^ and ETV6-NTRK3^G623R^ cells using viability assays. These data show that NTRK1^G595R^ is resistant to both larotrectinib and altiratinib (Fig. 2a, b). However, NTRK3^G623R^ retains partial sensitivity to altiratinib (IC_50_ ~ 240 nM) as compared to near-total resistance to larotrectinib (IC_50_ > 2000 nM). We performed immunoblotting to assess on-target NTRK inhibition from treated Ba/F3 cell lysates. These data suggest that altiratinib may indeed retain some inhibitory propensity against NTRK3^G623R^ as compared to NTRK1^G595R^ (Fig. 2c, d). Densitometry reveals that NTRK^G623R^ autophosphorylation is ~95% attenuated with 250 nM altiratinib (Fig. 2d, Supplementary Fig. 3b) whereas 250 nM larotrectinib inhibited NTRK^G623R^ by only ~41% (Fig. 2d, Supplementary Fig. 3a). Notably, loss of NTRK3 but not NTRK1 mutant fusion protein is observed with increasing concentrations of kinase inhibitor and this correlates with the extent of catalytic inhibition.

To determine the kinetics of catalytic inhibition-induced loss of ETV6-NTRK3 in Ba/F3 cells, and to compare this with TPM3-NTRK1, we treated stable Ba/F3 TPM3-NTRK1 and Ba/F3 ETV6-NTRK3 cell lines with 25 nM altiratinib for 2, 4, 6, and 24 h. These data show intact TPM3-NTRK1 levels even after 24 h of continuous exposure to 25 nM altiratinib as opposed to near complete and sustained loss of ETV6-NTRK3 after 2 h (Supplementary Fig. 3c, d). Densitometry from replicated experiments reveals that ≥50% of the total soluble pool of catalytically inactive ETV6-NTRK3 is lost in ≤2 h (Supplementary Fig. 3d) in contrast to TPM3-NTRK1. To test the reproducibility of this effect in an alternate cell model system, and to confirm that the observed protein downregulation is a consequence of selective NTRK3 catalytic inhibition, and not off-target activity of altiratinib, we transiently expressed ETV6-NTRK3 and ETV6-NTRK3^G623R^ mutant in HEK293A cells, and treated cells with altiratinib and the more selective inhibitor, larotrectinib (Supplementary Fig. 3e). Loss of ETV6-NTRK3 is observed with both the more selective type I inhibitor, larotrectinib and the multi-kinase type II NTRK inhibitor altiratinib, thus linking ETV6-NTRK3 downregulation to catalytic inhibition of NTRK3. The ETV6-NTRK3^G623R^ mutant has reduced affinity for both larotrectinib and altiratinib (Fig. 2), and since catalytic activity is inefficiently suppressed, protein loss is only minimally observed at the 75 nM concentration tested (Supplementary Fig. 3e). Based on these data, we propose a hypothetical model (Supplementary Fig. 3f) wherein sustained catalytic inactivity leads to the downregulation of NTRK3 fusion but not NTRK1 fusion, and future studies are needed to define the precise biochemical mechanisms regulating this phenomenon.

To gain structural insight into efficacy of altiratinib on these solvent front mutations, we performed molecular docking studies of the DFG-out conformation of NTRK1^G595R^ and NTRK3^G623R^ with altiratinib. Superimposition of NTRK1^G595R^ and NTRK3^G623R^, as well as individually docked structures are shown in Fig. 2e and Supplementary Fig. 3g and h, respectively. While NTRK1 and NTRK3 kinase domains have ~72 and 84% sequence identity and similarity, respectively, a few differences in the superimposed structures may account for altiratinib’s partial efficacy against NTRK3 but not NTRK1 mutant. For example, the angle of the predominant R595 rotamer in NTRK1 is oriented farther away from the binding pocket, as compared to that of R623 in NTRK3, albeit only modestly. Docking studies suggest a clear potential for steric clash with R595 in NTRK1, whereas this is less clear in case of NTRK3 R623. We propose that this subtle difference may accommodate altiratinib in the NTRK3^G623R^ binding pocket, potentially with lower affinity than NTRK3^wt^. Another striking difference lies in the modeled conformation of a loop (amino acids 603–623 in NTRK1 (green) and 631–662 in NTRK3 (magenta); however at this time it is unclear if or how this may contribute to the conformation and binding of TKI (Fig. 2e and Supplementary Fig. 3g, h).

### In vivo efficacy of the type II inhibitor altiratinib in a patient-derived NTRK3^G623R^ xenograft tumor model

**Fig. 3:**
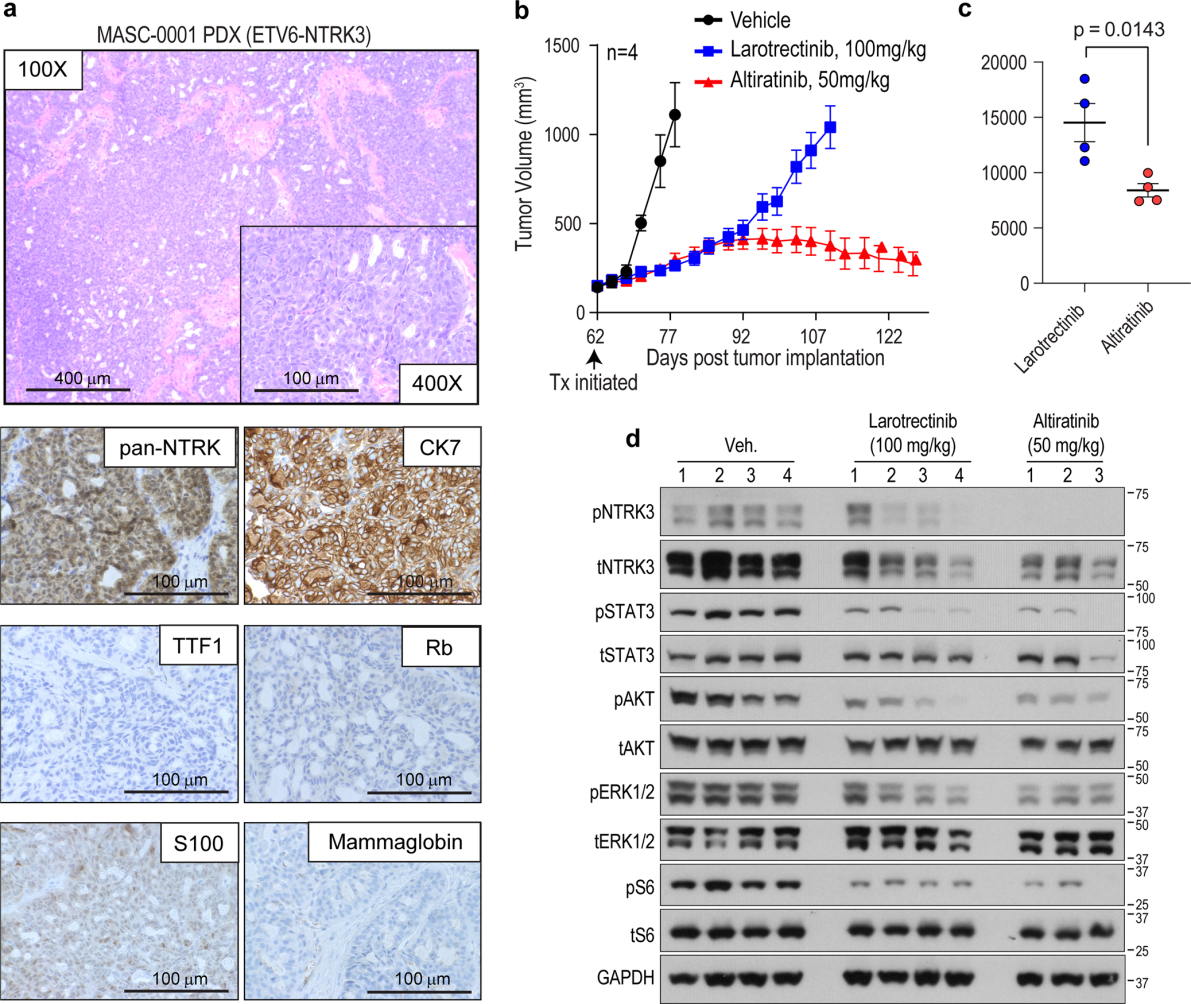
Altiratinib inhibits growth of ETV6-NTRK3^G623R^ patient-derived xenograft tumors. **a** Morphological and immunohistochemical characterization of patient-derived xenograft (PDX) that was established from tumor samples obtained from a MASC patient (MASC-0001) harboring the ETV6-NTRK3 fusion and who developed the G623R mutation after entrectinib treatment. **b** Mice bearing PDX tumors were treated with larotrectinib (100 mg/kg, twice daily (bis in die, (BID), shown in blue) or altiratinib (50 mg/kg BID, shown in red) as compared to vehicle-treated tumor-bearing mice (vehicle, BID, shown in black). Treatment (Tx) was initiated 62 days post implantation (black arrow) when tumor volume was ~100–200 mm^3^. Tumor volume measurements are shown as a function of time. **c** Area under the curve analysis compares the efficacy of larotrectinib and altiratinib tumors. Average ± standard error of means (SEM) is shown with statistical significance indicated by *p* value (unpaired Student’s *t*-test). **d** Immunoblot analysis of lysates generated from treated tumors (from (**b**)). Phosphorylation of ETV6-NTRK3 (pNTRK3), STAT3 (pSTAT3), AKT (pAKT), ERK1/2 (pERK1/2), and ribosomal protein S6 (pS6), as well as corresponding total levels of these proteins and GAPDH (loading control). “Veh.” indicates vehicle (DMSO) treatment. Numbers above blots represent separate tumors from individual mice with *n* = 4 for the vehicle and larotrectinib groups, and *n* = 3 for the altiratinib group. There was insufficient material from the 4th altiratinib-treated tumor for biochemical analysis.

Given that altiratinib showed partial inhibitory efficacy for NTRK3^G623R^ but not the paralog, NTRK1^G595R^ (Fig. 2), we interrogated its potential to inhibit tumor growth in vivo. For this, we employed a patient-derived xenograft (PDX) model that was generated from a patient diagnosed with *ETV6-NTRK3* fusion positive mammary analogue secretory carcinoma (MASC). The patient’s clinical history is outlined in Supplementary Fig. 4a. After an initial response to entrectinib, the patient had disease progression and next-generation sequencing by MSK-IMPACT revealed subclonal NTRK3^G623R^ solvent front mutation. Given our cell-based data indicating that altiratinib may retain some sensitivity for NTRK3^G623R^, we investigated its anti-tumor benefit in the MASC PDX model. We first performed histological characterization of this disease model (MASC-0001) (Fig. 3a). Hematoxylin and eosin-stained sample showed that the PDX tumor tissue closely resembled the previously published histology of the patient tumor^14^. Cells demonstrated granular eosinophilic cytoplasm and scattered vacuolation with cellular atypia of a mild form. Immunohistochemical staining for marker proteins showed negative staining for TTF1 and chromogranin, but positive for CK7 and S100 (Fig. 3a). The PDX sample was negative for mammaglobin, a typical positive marker for MASC. Loss of nuclear staining of RB reflected the truncating mutation in *RB1* found by NGS, while pan-NTRK immunostaining was strongly positive. The subclonal NTRK3^G623R^ mutation from PDX tumor tissue was validated by orthogonal DNA sequencing (Supplementary Fig. 4b). Animals bearing subcutaneous tumors were treated with larotrectinib (100 mg/kg, oral treatment (p.o.), twice a day (bis is die (bid)) or altiratinib (50 mg/kg, p.o., bid). While initial disease control was accomplished with larotrectinib treatment, the tumors started growing rapidly by day 96 (34 days of treatment), as compared to altiratinib treatment that controlled growth for more than two months, at which time the experiment was terminated (Fig. 3b). Comparison of the tumor volumes between the altiratinib- and larotrectinib-treated groups using area under the curve (AUC) analysis demonstrate that altiratinib significantly inhibited tumor growth (*p* = 0.0143, Fig. 3c). Biochemical analysis of tumors after oral administration of inhibitors shows that altiratinib treatment caused more robust downregulation of phosphorylation of NTRK3, STAT3, AKT, ERK1/2, and S6 than larotrectinib (Fig. 3d). As observed in the cell line studies described above, altiratinib treatment also lead to a more robust reduction in NTRK3 expression than larotrectinib (Fig. 3d). These data suggest that the anti-tumor efficacy of altiratinib in the MASC-0001 PDX model is likely due to inhibition of ETV6-NTRK3^G623R^. The treatments were well tolerated as measured by animal weight (Supplementary Fig. 4c). Based on previous pharmacokinetic and pharmacodynamics measurements, the 50 mg/kg BID treatment is estimated to result in free plasma concentration of altiratinib >250 nM for 24 h in mice^28,29^. At this time, clinical pharmacokinetic data are not available thus precluding definitive conclusion regarding the potential for altiratinib to suppress ETV6-NTRK3^G623R^ tumor growth in the clinical setting.

### Altiratinib effectively inhibits a subset of NTRK gatekeeper and xDFG kinase domain mutations

**Fig. 4:**
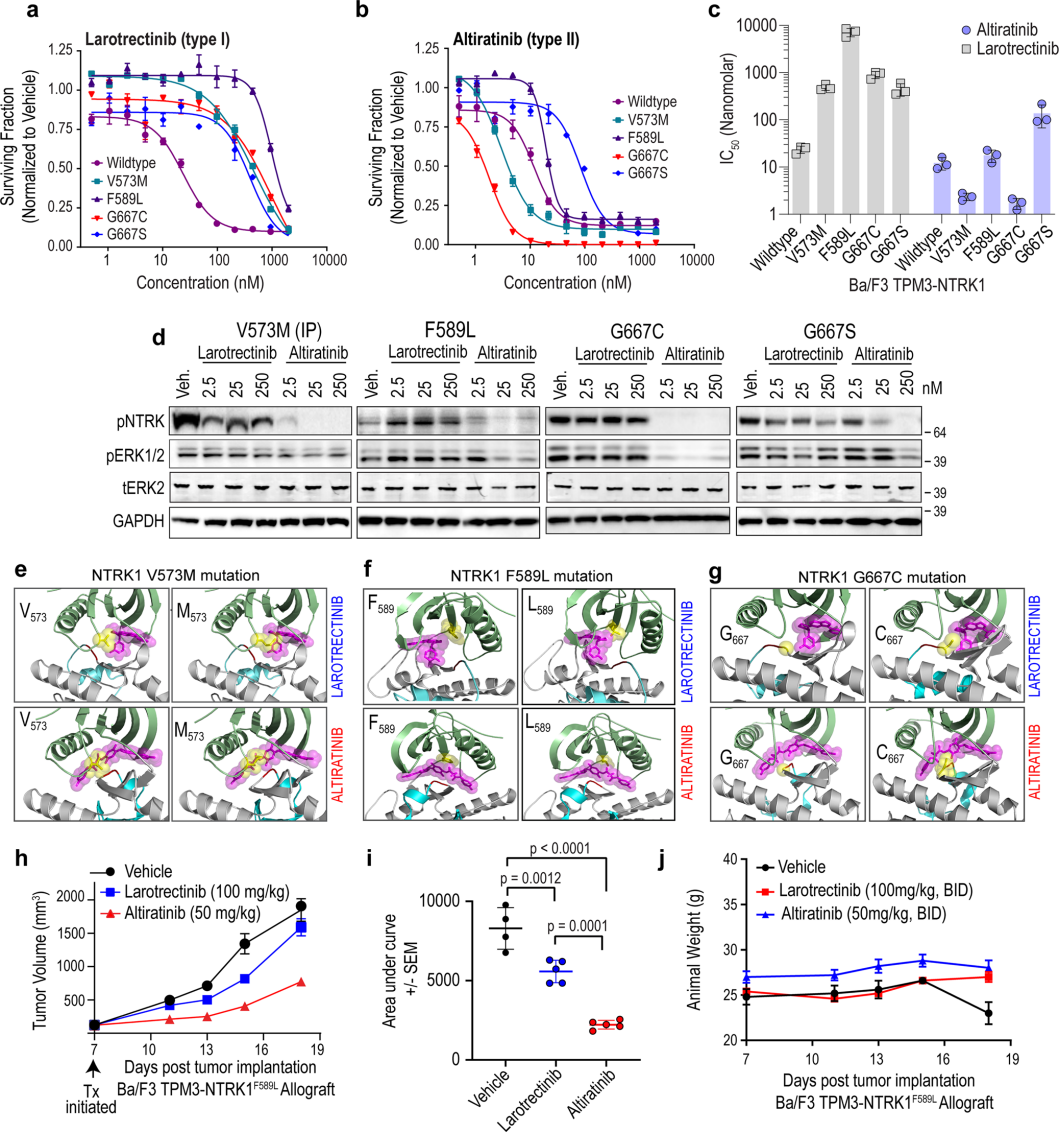
Inhibitory potential of larotrectinib and altiratinib on NTRK1 gatekeeper and xDFG mutations. Dose-response cell viability data comparing Ba/F3 cells expressing wildtype TPM3-NTRK1 or kinase domain mutants (V573M, F589L, G667C, and G67S) that were treated with larotrectinib (**a**) or altiratinib (**b**) for 72 h. Representative data are average ± standard error of means (SEM) from three independent replicates. **c** Column graph shows IC_50_ for indicated Ba/F3 cell lines treated with larotrectinib (teal bars) or altiratinib (gray bars). *Y*-axis depicts data on the log[10] scale. **d** Immunoblot analysis of TPM3-NTRK1 wildtype and mutants autophosphorylation (pNTRK), Erk1/2 (pErk1/2) phosphorylation, and corresponding total Erk2 and loading control (Gapdh) levels. “Veh.” indicates vehicle (DMSO) treatment. **e**–**g** Molecular docking of larotrectinib (top panel) or altiratinib (bottom panel) onto NTRK1 wildtype (left panel—wildtype amino acid) or mutant (right panel—mutated amino acid) structures. **h** Tumor volume as a function of time from mice bearing Ba/F3-TPM3-NTRK1^F589L^ allograft tumors that were treated with vehicle, larotrectinib (100 mg/kg, BID) or altiratinib (50 mg/kg, BID). Treatment (Tx) was initiated 7 days after implantation (black arrow). **i** Area under the curve analysis to compare the efficacy of larotrectinib and altiratinib in TPM3-NTRK1^F589L^ tumors to vehicle-treated tumors (average ± standard error of means (SEM)). Statistically significant differences as shown with asterisk and p values above respective columns. **j** Animal weight (grams (**g**)) measured during treatment with vehicle, larotrectinib and altiratinib.

Previous studies revealed that NTRK kinase domain mutations (V573M, F589L, G667C, and G667S) reduce affinity for larotrectinib and entrectinib^14,15,26^. We tested the comparative efficacy of larotrectinib (Fig. 4a) and altiratinib (Fig. 4b) against these NTRK mutations by employing the Ba/F3 cell model described above. As compared to the acquisition of resistance to larotrectinib, NTRK1 V573M, F589L, and G667C mutations retain partial to complete altiratinib-sensitivity (Fig. 4a, b). Specifically, altiratinib inhibited NTRK1 V573M, F589L, G667C, and G667S mutations with cell-based IC_50s_ of 2.8, 18.3, 1.8, and 93.5 nM, respectively, as compared to wildtype IC_50_ of 11.2 nM (Fig. 4b, c). Variable levels of TPM3-NTRK1 fusion are observed in Ba/F3 TPM3-NTRK1 wildtype, V573M, F589L, G595R, G667C, and G667S cell lines, however, the relative sensitivity (or resistance) is not correlated to overall oncogene dose (Supplementary Fig. 5a).

Intriguingly, the cysteine and serine substitutions at the G667 position in NTRK1 differentially affect altiratinib inhibitory potential. While both these amino acids have similar bulk, we believe that the unique sulfur-pi interactions, which stabilize binding of the inhibitor, that occur with the cysteine but not the serine residue, account of this difference. This is further explored in the structural modeling studies described below. Dose-dependent, on-target inhibitory action on NTRK autophosphorylation, and coupled modulation of downstream effector (ERK1/2 (RAS/MAPK pathway)) protein phosphorylation were confirmed using immunoblotting of cell lysates after treatment (Fig. 4d). These data are consistent with cell viability data in terms of the inhibitory potential of larotrectinib and altiratinib to suppress catalytic activity of NTRK1 wildtype and mutant kinases. Both inhibitors induced apoptosis in wildtype NTRK1 fusion-driven cells equivalently (Supplementary Fig. 5b). Consistent with the dose-response data, only altiratinib induced apoptosis in NTRK1^G667C^ cells at 2.5, 25, and 250 nM, and in and NTRK1^V573M^ and NTRK1^F589L^ at 25 and 250 nM (Supplementary Fig. 5b). The NTRK1^G667S^ substitution is relatively insensitive to altiratinib, with apoptosis induction only achieved with the 250 nM concentration.

We developed homology models based on existing crystal structures of NTRK1 (PDB 4AOJ; TrkA) and NTRK3 (6KZC; TrkC), and performed molecular dynamic (MD) simulation and molecular docking studies (Fig. 4e–g, Table 1, Supplementary Figs. 5c and 7). Larotrectinib and altiratinib were docked onto both the type I (DFG-in, ‘active’) and type II (DFG-out ‘inactive’) conformations of the NTRK1 kinase domain. Using this method, NTRK1^V573M^, NTRK1^F589L^, and NTRK1^G667C^ mutations were examined for their differential sensitivity to these two inhibitors. Docking studies reveal that larotrectinib interacts with the active form of NTRK1^V573M^ with sevenfold lower affinity as compared to NTRK1^wt^ (Table 1). Twelve of the 13 possible rotomers displayed steric clashes, and orient the thioether in M573 away from the aromatic rings in larotrectinib (Fig. 4e). This may explain the observed weakened binding of larotrectinib to NTRK1^V573M^. In contrast, the altiratinib IC_50_ for NTRK1^V573M^ (2.8 nM) is reduced as compared to NTRK1^wt^. Dissociation constants from molecular docking studies show that the affinity of altiratinib for NTRK1^V573M^ is reduced fourfold as compared to NTRK1^wt^, and parallel the cell-based observations. Based on modeling studies, we predict that this is likely due to the stability effect of S/π interactions between the thioether in methionine and the aromatic ring within the structure of altiratinib. The most favorable configuration places the sulfur atom at 4.4 Å from the ring plane (Fig. 4e and Supplementary Fig. 5c). Larotrectinib interacts with the active conformer of NTRK1^F589L^ with a ~27-fold lower affinity relative to NTRK1^wt^. The most predominant backbone-dependent rotomer (82%) displays pronounced steric clashes with the inhibitor. Meanwhile, altiratinib interacts with the inactive conformer of NTRK1^F589L^ with essentially unchanged affinity relative to its binding to NTRK1^wt^, and no major steric clashes are observed (Fig. 4f and Supplementary Fig. 5c).

NTRK1^G667C^ but not a serine substitution at the same position, NTRK1^G667S^, is hypersensitive to altiratinib; NTRK1^G667C^ and NTRK1^G667S^ both exhibit resistance to larotrectinib as compared to NTRK1^wt^. Molecular docking data reveal that larotrectinib interacts with the DFG-in form of NTRK1^G667C^ with a 40-fold lower affinity relative to NTRK1^wt^ (Table 1). The sulfur (S) atom in the cysteine is more than 6 Å away from the centroid of the closest aromatic ring in larotrectinib. In contrast to larotrectinib, altiratinib interacts with NTRK1^G667C^ with a ~2.5-fold higher affinity, and docking studies again show that the stabilization effect of S/π interactions^38^ between the sulfur atom in cysteine 667 and aromatic ring of altiratinib likely explain this higher affinity (Fig. 4g and Supplementary Fig. 5c). The serine substitution at the G667 position, on the other hand, does not permit these favorable interactions, and the functional cell-based data accordingly do not show sensitivity or hypersensitivity of altiratinib for this mutation (Supplementary Fig. 6a and Table 1). Functionally important sulfur−π interactions have been identified in the D2 dopamine receptor^39^ and the vital role played by these interactions in chemical and biological recognition and in drug development is well-recognized^40^.

To test if the in vitro efficacy of altiratinib to inhibit NTRK1 gatekeeper mutation translates to in vivo response, we performed murine allograft studies using Ba/F3-TPM3-NTRK1^F589L^ mutant cells. Tumor-bearing animals were treated with vehicle, larotrectinib (100 mg/kg p.o., BID) or altiratinib (50 mg/kg p.o, BID). Tumor growth as a function of time from vehicle, larotrectinib or altiratinib-treated animals is shown in Fig. 4h. Treatment with altiratinib caused a significant reduction in growth of NTRK1^F589L^ xenograft tumors compared to vehicle (AUC: vehicle −7937 ± 816, altiratinib −2219 ± 180, *p* < 0.0001) (Fig. 4i). Although larotrectinib treatment reduced tumor volume overall as assessed by AUC measurement (5579 ± 485, *p* = 0.02), by the end of the study, there was no significant difference between the larotrectinib and vehicle-treated groups (Fig. 4I). Inhibitor treatment was terminated on day 18 when the vehicle-treated tumors reached the maximum allowable size. As would be expected in patients treated with any targeted therapy, after treatment was discontinued, all tumors started re-growing, albeit recurrence in altiratinib-treated mice was slower than larotrectinib. The treatments were well tolerated as determined by body weight assessment (Fig. 4j).

### xDFG substitution G667C sensitizes TPM3-NTRK1 to inhibition by type II binding mode inhibitors

We further investigated the sensitization of NTRK1^G667C^ mutation to the type II inhibitors. Mutations at this position (G667) in the NTRK kinase domain are classified as xDFG with the ‘x’ indicating the position immediately amino-terminal to the DFG motif. We explored if NTRK1^G667C^ is generally resistant to type I inhibitors, and sensitized to type II inhibitors. For this, we used larotrectinib and entrectinib as representative ATP-competitive, type I inhibitors. A previous study reported that foretinib binds the DFG-out or type II conformation of NTRK1^41^. In addition, our previous work established that ROS1 kinase domain mutations that confer resistance to the type I inhibitor, crizotinib, retain sensitivity to the type II inhibitors, cabozantinib, and foretinib^24^. ROS1 and NTRK tyrosine kinase domains have 86% amino acid similarity, and 38% identity. Based on this, we hypothesized that similar to altiratinib, foretinib and, the FDA-approved multi-kinase inhibitor, cabozantinib that is closely related to foretinib, may be effective against xDFG mutations.

**Fig. 5:**
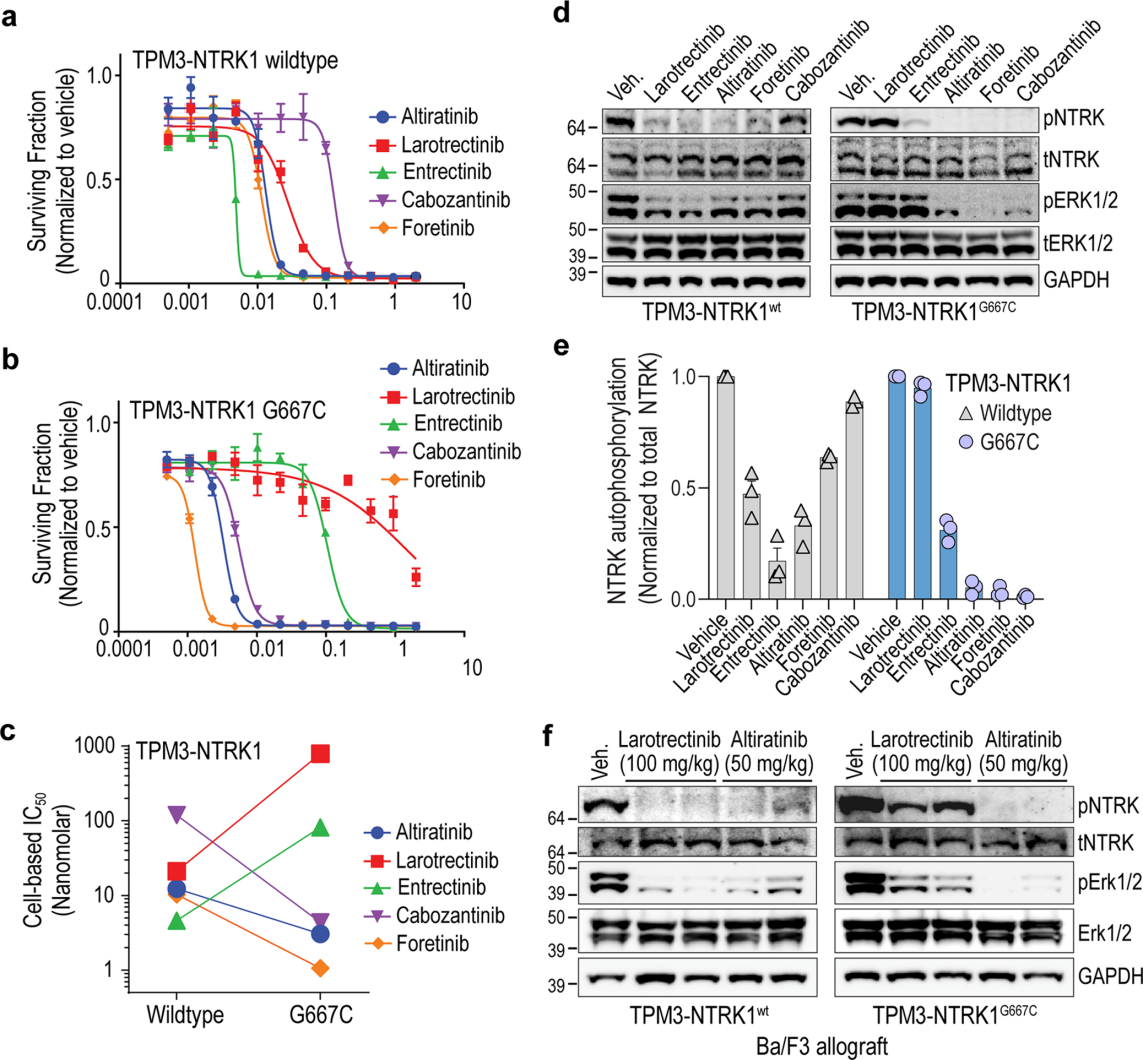
The xDFG mutation NTRK1^G667C^ sensitizes the kinase to type II inhibitors. Dose response cell viability data comparing the activity of indicated inhibitors in Ba/F3 TPM3-NTRK1 (**a**) or TPM3-NTRK1^G667C^ (**b**) cell lines after 72 h treatment. Data are representative of four independent experiments for larotrectinib, entrectinib, and altiratinib, and three independent experiments for cabozantinib and foretinib. Average ± SEM from three replicates shown. **c** Scatter plot compares IC_50_ of indicated inhibitor for Ba/F3 TPM3-NTRK1 wildtype versus G667C mutant cells. **d** Immunoblot analysis of TPM3-NTRK1^wt^ (wildtype) TPM3-NTRK1^G667C^ autophosphorylation (pNTRK) and effector protein, Erk1/2 phosphorylation (pErk1/2) and corresponding total NTRK (tNTRK) and total Erk1/2 (tErk1/2) as well as loading control (Gapdh) levels from Ba/F3 cells treated with indicated inhibitors. “Veh.” indicates vehicle (DMSO) treatment. Data are representative of three independent experiments. **e** Densitometry of pixel intensity to show relative auto-phosphorylation of TPM3-NTRK1 wildtype (red columns) and G667C (blue columns) mutant from three experiments. Average ± SEM are shown. Individual values are shown as symbols at the top of the column: wildtype—triangles and G667C—circles. **f** Immunoblot analysis from Ba/F3 allograft tumors with lysates harvested 3 h after a single dose of larotrectinib (100 mg/kg) or altiratinib (50 mg/kg) treatment. TPM3-NTRK1^wt^ (wildtype) TPM3-NTRK1^G667C^ autophosphorylation (pNTRK) and effector protein, Erk1/2 phosphorylation (pERK1/2) and corresponding total NTRK (tNTRK) and total Erk1/2 (tErk1/2) as well as Gapdh (loading control).

We compared these type I and type II inhibitors for their inhibitory efficacy on NTRK1^wt^ and discovered that with the exception of cabozantinib (IC_50_ = 73.1 nM), larotrectinib, entrectinib, altiratinib and foretinib blocked cell growth with IC_50_ ≤ 30 nM (Fig. 5a, Supplementary Fig. 6). The ~9-fold difference in cell-based inhibitory activity of foretinib versus cabozantinib for wildtype NTRK1 was unexpected given their nearly identical molecular structures^24^. Performing dose response cell viability assays with TPM3-NTRK1^G667C^ cells revealed uniform resistance of this mutant kinase to the type I inhibitors (larotrecintib and entrectinib), and notable sensitization to all type II inhibitors tested (altiratinib, cabozantinib and foretinib). Specifically, 50% growth inhibition of Ba/F3 TPM3-NTRK1^G667C^ required 37- and 27-fold higher concentrations of larotrectinib and entrectinib, respectively, as compared to TPM3-NTRK1^wt^ cells (Fig. 5b, c, and Supplementary Fig. 6). In contrast, the type II inhibitors, altiratinib, cabozantinib and foretinib exhibited 2-, 17- and 10-fold reduced IC_50_, respectively, for the same mutation. Summary of the type I and type II inhibitors tested on wildtype and mutant NTRK fusion cell lines is in Supplementary Fig. 6.

Root mean square deviation (RMSD) data from MD simulations reveal that while the total kinase structure of NTRK1 is essentially stable in both the DFG-in and DFG-out conformations of wildtype and mutant protein, the activation (A) and P-loops exhibit larger degree of flexibility in the DFG-out conformation, as compared with the DFG-in conformation (Supplementary Fig. 7). These data suggest local alterations in conformation likely have a differential impact on the binding of type I versus type II inhibitor for a given mutation. Dissociation constants (d.c.) derived from docking studies (described in methods) concord with cell-based IC_50_ data in that altiratinib and foretinib consistently display high affinity (lower d.c.) for the inactive (DFG-out) NTRK1 (Table 1). In contrast, larotrectinib and entrectinib display preference for the active (DFG-in) conformation of TrkA. Consistent with the hypersensitivity of Ba/F3 TPM3-NTRK1^G667C^ but not the TPM3-NTRK1^G667S^ cells to the type II inhibitors (altiratinib, foretinib and cabozantinib), the dissociation constants for the cysteine substitution are lower than for the serine substitution at the 667 position, demonstrating higher affinity (Table 1). Intriguingly, some additional nuances were observed in terms of cabozantinib and foretinib. Even though these inhibitors are nearly identical in structure, cabozantinib is a poor inhibitor of wildtype TrkA as compared with foretinib. In contrast, foretinib is not only an effective inhibitor of wildtype (DFG-out) NTRK1, but also exhibits a higher affinity for the serine substitution rather than the cysteine substitution at the G667 position (Table 1).

We performed immunoblotting of cell extracts isolated from Ba/F3 TPM3-NTRK1^wt^ or TPM3-NTRK1^G667C^ cells after treatment with 15 nM inhibitor as indicated. Larotrectinib, entrectinib, altiratinib, and foretinib inhibited NTRK1^wt^ autophosphorylation (pNTRK1) effectively, whereas cabozantinib was infective at this concentration, consistent with dose-response cell viability data (Fig. 5d). Treatment of NTRK1^G667C^ expressing cells with altiratinib, foretinib and cabozantinib inhibited pNTRK and activation of downstream MAPK1/3 (pERK1/2). Densitometry of pNTRK1 normalized to total NTRK1 levels from replicate experiments is shown in Fig. 5e. Finally, we compared the ability of larotrectinib and altiratinib to inhibit pNTRK1^wt^ and pNTRK1^G667C^ and attenuate downstream effector signaling in vivo. Mice bearing Ba/F3 TPM3-NTRK1^wt^ or TPM3-NTRK1^G667C^ allograft tumors were treated with a single dose of either larotrectinib (100 mg/kg, p.o.) or altiratinib (50 mg/kg, p.o.) and tumors were harvested after 3 h for biochemical analysis. Concordant with in vitro studies, altiratinib effectively inhibited intra-tumoral phosphorylation of NTRK3^G667C^ and Erk phosphorylation (Fig. 5f).

## Discussion

Advances in next-generation sequencing-based cancer diagnostics has facilitated the discovery of *NTRK* rearrangements in a diverse array of malignancies^2^^,^^5,6,42^. Importantly, concomitant drug development efforts and innovations in clinical trial design have led to the rapid advancement and approvals of two NTRK inhibitors, larotrectinib and entrectinib, by the Food and Drug Administration in the US^3,6^. Over the last few years, rationally guided drug design has resulted in the development of several second-generation type I compounds such as repotrectinib, selitrectinib and DS-6051b that retain potency for solvent front resistant mutations (NTRK1^G595R^ and NTRK3^G623E/R^) in pre-clinical studies and in some cases, in patients^6,26,27^. The global adoption of molecular targeted NTRK therapy is rapidly growing, and clinical trials or treatment data show impressive patient responses in some patients. However, the emergence of on-target or bypass resistance pathways continues to pose formidable clinical challenges that hinder long-term durability in many patients.

Our data demonstrate that the xDFG mutations are resistant to the first generation type I inhibitors, and we postulate that these xDFG mutations will continue to pose a therapeutic liability for the newer type I inhibitors as well. Thus, mutations at this position may eventually account for a substantial portion of on-target resistance, including to selitrectinib, taletrectinib, and repotrectinib. Newer, combination or alternate pharmacologic strategies, including repurposing existing FDA-approved type II inhibitors, such as cabozantinib, or the development of more selective type II NTRK inhibitors may be necessary to gain complete control over the xDFG mutations.

A limitation of our study is that while molecular docking studies using homology models of kinase structures can reveal valuable structural data, this approach is not a complete substitute for crystallographic studies. In the biophysical studies that utilize purified protein, the mutant kinases may adopt slightly different conformation(s) that we cannot accomplish in silico, due to either autophosphorylation during the process of protein production or other post-translational modifications. However, our study here lays the foundation for future determination of inhibitor bound NTRK mutant structures using X-ray crystallography, and for the rational design of novel type II NTRK inhibitors to mitigate resistant mutations.

An unexpected finding was that treatment of cells with larotrectinib or altiratinib results in drastic loss of observable ETV6-NTRK3 fusion protein in the soluble protein fraction, but not of TPM3-NTRK1. The tyrosine kinase domains of NTRK1 and NTRK3 are 78% identical (amino acid level). Thus, the post-translational modifications or recruitment of degradation-associated proteins likely occurs outside the kinase domain, with carboxy- or juxtamembrane domains as potential regions of interest. Tognon et al. showed that the E3 ubiquitin ligase KPC1 is recruited to dephosphorylated ETV6-NTRK3 fusion followed by its poly-ubiquitination^43^. In their experiments, knockdown of KPC1 rendered NTRK3 fusion resistant to inhibitor-induced degradation. Based on their studies with the compound BMS-536924, they proposed a model wherein IGF1R activity controls the stability of ETV6-NTRK3 fusion protein. However, BMS-536924 is a relatively promiscuous early generation kinase inhibitor with activity against multiple kinases. The results with BMS-536924 may actually be reflecting its inhibitory activity against NTRK itself, rather than IGF1R. This is confirmed by the evaluation of KINOMEscan data that shows that BMS-536924 is slightly more effective at inhibiting NTRKs than IGF1R (http://lincs.hms.harvard.edu/db/datasets/20112/main). In contrast, larotrectinib is a highly selective NTRK inhibitor, with no efficacy against IGF1R. Given our observation of ETV6- NTRK3 downregulation after larotrectinib treatment, we conclude that the stability of this fusion protein is intrinsically regulated by alterations in post-translational modifications that are linked to autophosphorylation state. Specifically, taking the findings of Tognon et al.^43^ into account, we hypothesize that when NTRK3 is auto-phosphorylated, it adopts a conformation that is refractory to interaction with KPC1. Inhibitor-induced dephosphorylation may alter NTRK3 conformation to promote the recruitment of KPC1, and subsequent degradation. Future experiments employing genetic and pharmacologic approaches may offer deeper insight into this regulatory mechanism and open additional avenues to therapeutically downregulate the NTRK3 fusion driver using allosteric inhibitors.

Broadly, our data show that there are nuances pertaining to the structure and regulation of wildtype versus mutant NTRK kinases, and potentially between NTRK1 and NTRK3 as well. These conformational and regulatory differences dictate variable sensitivity to type I or type II inhibitors. To achieve durable patient responses, it may be beneficial to have a portfolio of clinically developed NTRK inhibitors that exhibit distinct binding properties in order to mitigate resistance depending on the NTRK fusion and the specific kinase domain mutation.

## Methods

### Inhibitors and plasmids

Altiratinib (DCC-2701), entrectinib (RXDX-101), and larotrectinib (LOXO-101) used in pre-clinical studies were provided by Deciphera Pharmaceuticals. Cabozantinib (XL-184) and foretinib (XL-880) were purchased from Selleckchem. The plasmids pMSCV-IRES-mCherry TPM3-NTRK1 and pMSCV-IRES-Puromycin ETV6-NTRK3 were generous gifts from Dr. Suzanne Baker (St. Jude Children’s Hospital), and Drs. Poul Sorensen and Cristina Tognon (University of British Columbia), respectively.

### Cell culture

Ba/F3, and HEK293A cells were purchased from American Type Culture Collection and Thermo-Fisher Scientific, respectively. Ba/F3 cells are a murine interleukin-3 (IL-3)-dependent pro-B cell line that become IL-3-independent when the cells are transformed with oncogenes. HEK293A is a subclone of the HEK293 cells that have improved adherence and flatter morphology. Parental Ba/F3 cells were cultured in complete medium [RPMI1640 supplemented with 10% (v/v) fetal bovine serum (FBS), 2 mmol/L L-glutamine, penicillin, streptomycin] and 2 ng/mL recombinant murine IL-3. HEK-293T cells were cultured in complete medium [DMEM, with 10% (v/v) BGS, L-glutamine, penicillin/streptomycin]. All cells were tested for mycoplasma every 6 months.

### Generation of cell lines and transformation assays

The *TPM3-NTRK1* and *ETV6-NTRK3* mutants were generated using site-directed mutagenesis following manufacturer’s protocol (Agilent). Platinum-E cells (Cell Biolabs, Inc.) were transfected with pMSCV-IRES-mCherry TPM3-NTRK1 or pMSCV-IRES-Puromycin ETV6-NTRK3 wildtype and mutant constructs using DNA transfection reagent from Biotool to generate replication incompetent, ecotropic retrovirus. The retroviruses produced by this method were used to transduce Ba/F3 cells. Ba/F3 cells were maintained at densities of 0.5–1.5 × 10^6^ cells/mL and infected with retrovirus encoding native or mutant versions of TPM3-NTRK1 and ETV6-NTRK3. The selection was performed either by cell sorting using FACS Aria cell sorter (BD Biosciences) for mCherry expression in TPM3-NTRK1 transduced cells or puromycin treatment in ETV6-NTRK3 transduced cells. To generate transformed, IL-3 independent, stable Ba/F3 cell lines, the cells were triple washed with IL-3 free complete medium and propagated. The cells that grew out after IL-3 withdrawal were maintained in a complete medium without IL-3 and used for in vitro assays.

### Inhibitor screening and cell viability assays

Inhibitors were prepared as 1 mmol/L stocks in DMSO prior to each experiment. Inhibitors were distributed at 2× concentration using a D300 Digital Dispenser (Hewlett-Packard) capable of accurately administering very small volumes (10 pL–150 nL) into 384-well plates preloaded with 25 µL per well of complete medium. Ba/F3 cells expressing TPM3-NTRK1 wildtype or mutant, or ETV6-NTRK3 wildtype or mutant constructs were seeded (750 cells per well; 25 µL) into drug plates using a Multidrop Combi Reagent Dispenser (Thermo Scientific), and plates were incubated for 72 h at 37 °C, 5% CO_2_. Viability was measured using a methanethiosulfonate (MTS)-based assay (CellTiter96 Aqueous One Solution; Promega) and read on a Biotek Synergy 2 plate reader. Proliferation experiments were performed three independent times where each condition was assayed in triplicate determinations. Data were normalized using Microsoft Excel and GraphPad Prism software determined the 50% growth inhibitory concentration (IC_50_) using a non-linear curve fit equation, modified using previously described parameters (1).

### Sanger sequencing validation of endogenous NTRK3 mutation

DNA was extracted from the cells using an AllPrep RNA/DNA Mini kit (Qiagen, Valencia, CA) according to the manufacturer’s instructions. cDNA (500 ng RNA) was synthesized using SuperScript VILO cDNA Synthesis Master Mix (ThermoFisher Scientific). For RT-PCR reactions, PCR amplification was preformed using AmpliTaq Gold 360 Mastermix (ThermoFisher Scientific) under the following conditions: 95 °C for 5 min, 36 cycles of denaturation at 95 °C for 15 s, annealing at primer-dependent temperature (Supplementary Table 1) for 30 s and extension at 72 °C for 30 s, with a final extension of 7 min at 72 °C. No template reactions were performed as negative controls. Primer sets were purchased from Applied Biosystems (Foster City, CA). PCR amplification was conducted on an ABI StepOne Real-Time PCR Instrument (Applied Biosystems). PCR amplicons were extracted using PCR purification kit (Qiagen, Valencia, CA), and the G623R mutation was confirmed by Sanger sequencing.

### Immunoblotting

Isogenic Ba/F3 cell lines expressing TPM3-NTRK1, TPM3-NTRK1^V573M^, TPM3-NTRK1^F589L^, TPM3-NTRK1^G667C^, TPM3-NTRK1^G667S^, TPM3-NTRK1^G595R^, ETV6-NTRK3, or ETV6-NTRK3^G623R^ cell lines were cultured in the absence or presence of inhibitors. Cells were treated with the indicated concentrations of inhibitors for 2 h, pelleted, washed once in ice-cold PBS, and lysed in 200 μL lysis buffer (Cell Signaling Technology (CST)) supplemented with 0.25% deoxycholate, 0.05% SDS, and protease and phosphatase inhibitors. Protein concentration was determined using the PierceTM BCA Protein Assay kit (ThermoFisher Scientific). Protein was extracted with Laemelli sample buffer for 10 min at 75 °C. Equal quantities of extracted lysates were run on 4–12% BIS-TRIS precast gradient gels (Criterion; Bio-Rad). Proteins were transferred to Nitrocellulose membranes (Millipore) and probed with an anti-phospho-Trk antibodiy that recognizes phospho-TrkA (Y674/675) and phosphor-TrkB (Y706/707) [4621, 1:1000; CST], pan-TRK (clone A7H6R) [3266, 1:1000; CST], phospho-p44/42 MAPK (T202/Y204)[9101, 1:1000; CST], total p44/42 MAPK (ERK1/2)[4696, 1:1000; CST], and GAPDH [clone AM4300, 1:5000; Invitrogen]. Blots were imaged using the Bio-Rad ChemiDoc imaging station according to the manufacturer’s protocol for immunoblot detection with use of horseradish peroxidase-conjugated secondary antibodies.

### Histology and immunohistochemistry

Hematoxylin- and eosin-stained slides from the PDX were closely inspected and compared to the clinical record of the patient. Unstained tissue sections on slides were deparaffinized and rehydrated; after antigens were retrieved, immunohistochemistry staining for the following antibodies was performed: pan-TRK (Abcam, clone EPR17341), TTF1 (Abcam, EPR5955), CK7 (Dako, clone OV-TL 12/30), S100 (Dako, rabbit polyclonal), chromogranin (Dako, polyclonal rabbit), mammaglobin (Dako, clone 304-1A5), RB (Leica Biosystems, clone 13A10). A relevant positive control tissue was concurrently stained for each antibody to ensure proper staining.

### Homology modeling

The X-ray structures of the kinase domains of the high-affinity NTRK1 (TrkA, (4AOJ; Chain A) and NTRK3 (TrkC) (6KZC; Chain A) were used as the template for building the open and closed conformers of NTRK1/TrkA and NTRK3/TrkC, respectively. The open and closed loops were identified using a BLAST search against the PDB, and then alignments were subsequently scored and optimized. Side-chain rotomers were modeled using backbone-dependent probabilities and optimized through MD and knowledge-based force fields using YASARA software^44^. The resulting models were optimized for hydrogen bonding, refined using MD simulations, and ranked. Residue specific quality graphs were calculated for each model and a final hybrid model was developed through an iterative process, replacing poorly scoring regions in the best model with the corresponding regions from other models. The stereo-chemical properties of the homology models were verified using the PROCHECK module of the PDBSum server^45^, which examines protein quality based on parameters such as percentage residues lying in favored and allowed regions, the number of glycine and proline residues and orientation of dihedral angles including phi (φ) and psi (*ψ*) and backbone conformation. The VERIFY3D server^45^ was used to check the compatibility of atomic models (3D) with its own primary amino acid sequences (1D).

### MD simulations

To assess the potential functional impact of the variants on the active and inactive conformations of TrkA, the system was simulated atomistically for 25 ns using the YASARA software package^46^ under an NPT ensemble with the AMBER14 force field^47^, with a timestep of 5.0 fs. Simulation conditions were conducted with periodic boundaries, at 0.9% NaCl concentration by mass, pH 7.4, 298 K, at atmospheric pressure. The water model employed was TIP3 equivalent. Snapshots were saved every 250 ps. The RMSD for the C-alpha atoms between the initial minimized structure and individual poses during the simulation were calculated for the A-loop (residues 514–524), P-loop (residues 669–689), and the entire kinase model (RMSD total). The data were plotted using Prism GraphPad software.

### Molecular docking

Flexible molecular docking protocols normally require high-resolution X-ray structures of the receptor. However, given the high sequence similarity between TrkA (NTRK1) and known kinase structures, and because the active sites are essentially conserved between templates and target, we have confidence that the homology models are of sufficient quality to perform comparative docking studies for the different inhibitors. To minimize the influence of less conserved regions on the docking, inhibitor binding was limited to the 7 Å simulation cell around the active site. The hydrogen-bonding network was optimized at a pH of 7.4 to ensure appropriate protonation states of side-chains. An analysis of ligands to determine the internal degrees of freedom was performed using Yasara run_ensemble_dock script. Receptor flexibility is considered by creating a receptor ensemble with alternative high-scoring solutions of the side-chain rotamer network at a temperature of 298 K. Docking was performed using VINA^48^ with default parameters. The protocol initiates searches from random locations with random ligand conformations, and automatically clusters the results by walking through the poses in order of worsening binding energy and includes all poses with a RMSD value that is <5 Å within a single cluster. This setup was done with the YASARA molecular modeling program^46^ and the best hits from 160 docks for a given pose of the MD simulation were analyzed. The lowest dissociation constants from the inhibitor simulation were averaged for every snapshot of the MD and these data are presented in Table 1.

### Generation of patient-derived model and in vivo efficacy studies

Tissue samples were collected under an institutional IRB-approved biospecimen collection protocol. All mice were cared for in accordance with guidelines approved by the Memorial Sloan Kettering Cancer Center Institutional Animal Care and Use Committee and Research Animal Resource Center and animals were monitored daily. Two core biopsies were collected from a lung metastasis of a MASC expressing *ETV6-NTRK3* fusion. The patient was previously described^14^. The tumor samples were minced, mixed with matrigel, and implanted subcutaneously in the right flank of a female *NOD/SCID* gamma (NSG, Jackson Laboratory, Bar Harbor, ME) mouse (6-week old) to generate the patient-derived xenograft (PDX)^49^. PDX tumors were serially transplanted three times prior to being used for this study. The PDX model was named MASC-0001. For efficacy studies, fresh PDX tumor samples (~3 mm^3^) were minced, mixed with matrigel, and implanted into the subcutaneous flank of six-week-old female NSG mice. Xenografts of KM-12 cells and transformed Ba/F3 were established by implanting 5 million cells mixed with 50% serum-free RPMI and 50% (v/v) matrigel into a subcutaneous flank of female athymic nude mice (6-week old) (Envigo, Indianapolis, Indiana). Xenografts of transformed NIH-3T3 cells was generated by implanting 2.5 million cells mixed with 50% serum-free RPMI and 50% (v/v) matrigel into a subcutaneous flank of female SCID mice (6-week old) (Envigo, Indianapolis, Indiana). Once tumors reached ~100 mm^3^ volume for both Ba/F3 (day 7) and PDX model (day 62), mice were randomly assigned to groups of five (Ba/F3 study) or four (PDX study) and treatment initiated with vehicle, larotrectinib (100 mg/kg, BID, oral gavage) or altiratinib (50 mg/kg, BID, oral gavage). In the Ba/F3 experiment, one mouse died before the last measurement in the vehicle group leaving only four animals at end of the study. For NIH-3T3 and KM-12 xenograft models (10 mice per group), treatment began when the average tumor burden was 114 or 150 mg, respectively (day 4 for KM-12 and day 5 for NIH-3T3), with the doses of altiratinib indicated in figure legends. Larotrectinib was resuspended in labrafac and altiratinib was resuspended in 0.4% hyroxypropylmethylcellulose (HMPC). Tumor size and animal weight were measured twice weekly, and tumor volume was calculated using the empirical formula: volume = length × width^2^ × 0.52. Animals were sacrificed when tumors reached 1000–1250 mm^3^ or if there were signs of sickness such as significant weight loss. All tumors were collected 3 h after the last dosing. For the Ba/F3 TPM3-NTRK1^wt^ and Ba/F3 TPM3-NTRK1^G667C^ studies that were designed to analyze target engagement by the small molecules in vivo, mice were treated with a single dose of larotrectinib (100 mg/kg) and altiratinib (50 mg/kg) and then tumors harvested 3 h later. Statistical analyses were performed using Graphpad Prism V8. Unpaired Student’s *t*-test was used to compare area under curve or tumor volume and *p* < 0.05 was considered significant.

### Statistics and reproducibility

In vitro studies represent three independent experiments in which each condition was assayed in triplicate determinations. Graphad Prism software was used to analyze data by non-linear regression to generate IC_50_ values and curve fitting. All data are presented as the mean ± SEM of all experiments. Western blotting was conducted on independently prepared samples three times. For animal studies, each group consisted of four or five mice. The tumor volumes presented represent the mean ± SEM of all tumors in the group. Area under curve analysis was used to compare groups and unpaired Student’s *t*-test was used to determine significance. *P* < 0.05 was considered significant.

### Reporting summary

Further information on research design is available in the Nature Research Reporting Summary linked to this article.

## Supplementary information

Supplementary Information

Reporting Summary

## Data Availability

Data from these studies (Figs. 1–5, Table 1, Molecular Docking) have been uploaded to  Figshare.com: 10.6084/m9.figshare.c.5203085.v3. Structural modeling was based on existing NTRK1 (TrkA) and NTRK3 (TrkC) kinase structures: TrkA (Chain A was used)—https://www.rcsb.org/structure/4aoj and TrkC (Chain A was used)—https://www.rcsb.org/structure/6KZC.
