# Supplementary material for: NTRK kinase domain mutations in cancer variably impact sensitivity to type I and type II inhibitors
Source: Commun Biol. 2020 Dec 16;3:776. doi: 10.1038/s42003-020-01508-w (PMC7745027; doi:10.1038/s42003-020-01508-w)
Supplement: Supplementary file 1 — Supplementary Information [file 42003_2020_1508_MOESM1_ESM.pdf]

SUPPLEMENTARY FIGURES AND FIGURE LEGENDS

NTRK kinase domain mutations variably impact sensitivity to type I and type II inhibitors

Romel Somwar[1,2], Nicolle E. Hofmann[3], Bryan Smith[4], Igor Odintsov[1,2], Morana Vojnic[1,2], Irina Linkov[1], Ashley Tam[3], Inna Khodos[5], Marissa S. Mattar[5], Elisa de Stanchina[5], Daniel Flynn[4], Marc Ladanyi[1,2], Alexander Drilon[6], Ujwal Shinde[7#], and Monika A. Davare[3#*]

1. Department of Pathology, Memorial Sloan Kettering Cancer Center, New York, NY, USA
2. Human Oncology and Pathogenesis Program, Memorial Sloan Kettering Cancer Center, New York, NY, USA
3. Department of Pediatrics, Oregon Health & Science University, Portland, OR, USA
4. Deciphera Pharmaceuticals, 200 Smith Street, Waltham, MA, USA
5. Antitumor Assessment Core Facility, Memorial Sloan Kettering Cancer Center, New York, NY, USA
6. Thoracic Oncology Service, Division of Solid Tumor Oncology, Department of Medicine, Memorial Sloan Kettering Cancer Center, New York, NY, USA
7. Department of Chemical Physiology and Biochemistry, Oregon Health & Science University, Portland, OR, USA

[#] Equal Contribution

*Correspondence: davarem@ohsu.edu

Address: Mail Code L321, OHSU, 3181 SW Sam Jackson Park Rd, Portland, OR 97239

Phone: 503-494-5056; Fax: 503-418-5044

Supplementary Figure 1

Ba/F3 Parental cells cultured with requisite cytokine mIL-3

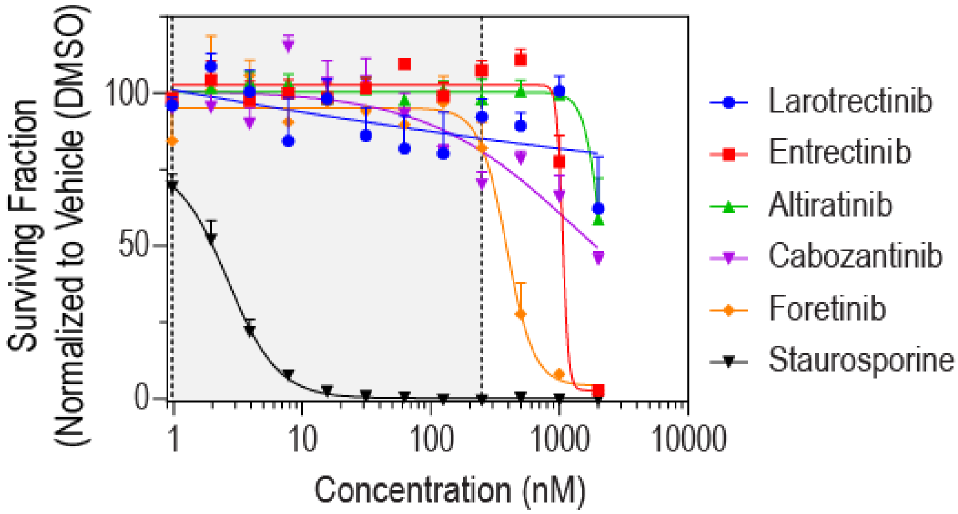

Gray box indicates range of concentration of larotrectinib, entrectinib, altiratinib, cabozantinib and foretinib required to inhibit wildtype or mutant NTRK kinases.

Staurosporine, a broad spectrum, multi-kinase inhibitor serves as positive control for cell death in Ba/F3 parental cells.

Supplementary Figure 1. Cell viability of Ba/F3 Parental cells. Dose response cell viability assay of Ba/F3 parental cells treated with indicated doses of larotrectinib, entrectinib, altiratinib, cabozantinib, foretinib and staurosporine as positive cell death control given its broad-spectrum kinase inhibition. Media contained murine interleukin-3 as this is a requisite cytokine for the survival of non-transformed Ba/F3 cells. Average ± standard error of means from three replicates per inhibitor and per concentration are shown.

Supplementary Figure 2

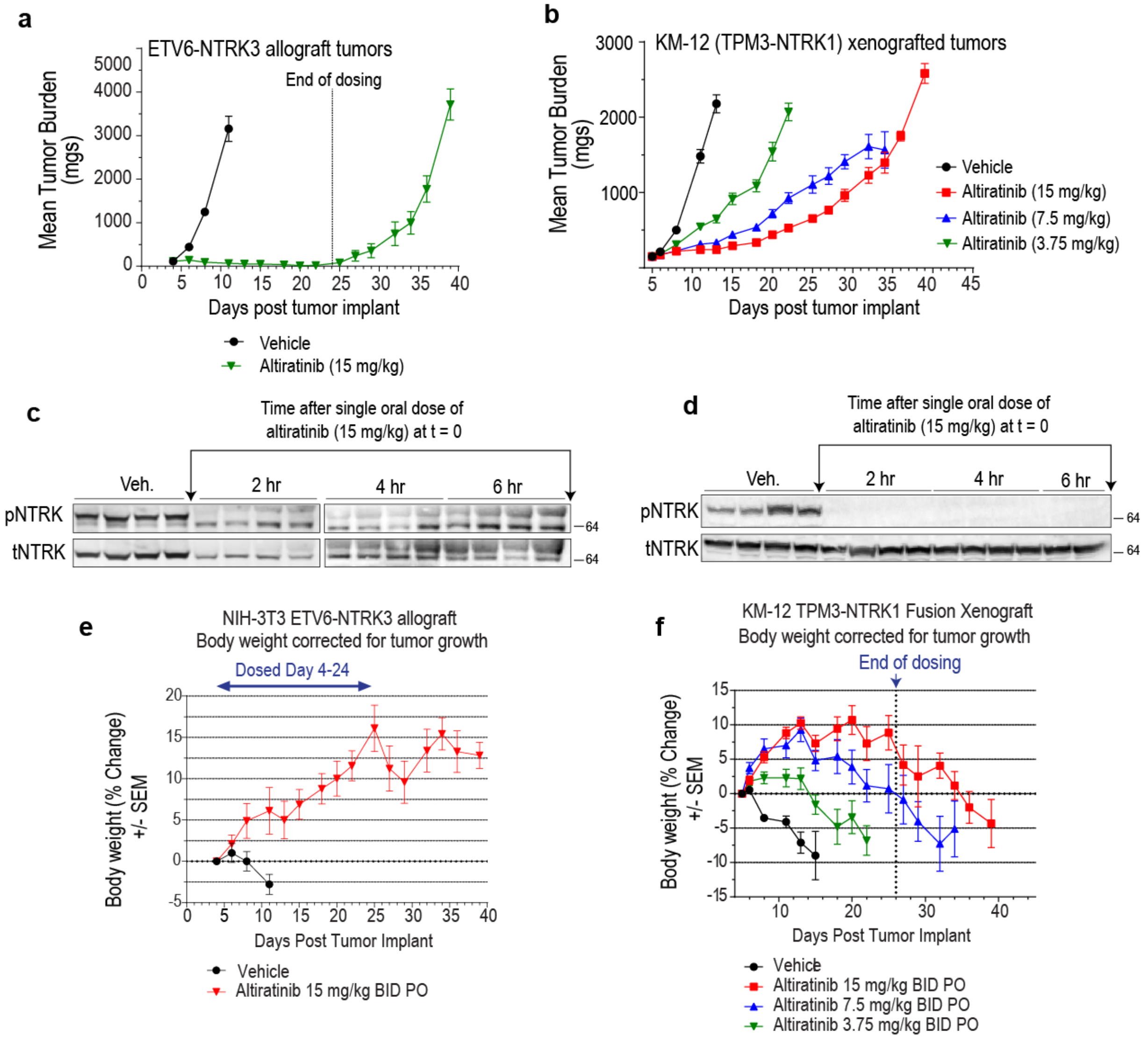

Supplementary Figure 2. In vivo efficacy of altiratinib in allograft and xenograft models of NTRK fusion driven tumors. a. Tumor volume (mm^3) as function of time in NIH3T3 ETV6-NTRK3 allograft tumors treated with vehicle or altiratinib (15 mg/kg) via oral gavage. Dotted line at day 24 indicates end of oral dosing. **b.** Tumor volume (mm^3) as function of time in KM-12 (TPM3-NTRK1 harboring human colorectal cancer cell line) xenografted tumors treated with vehicle or altiratinib (3.75, 7.5 or 15 mg/kg) via oral gavage. **c.** Immunoblot analysis of NTRK3 autophosphorylation (pNTRK) and total NTRK3 (tNTRK) fusion levels in tumor lysates from NIH3T3 ETV6-NTRK3 allografts. **d.** Immunoblot analysis of NTRK1 autophosphorylation (pNTRK) and total NTRK1 (tNTRK) fusion levels in tumor lysates from KM-12 (TPM3-NTRK1) xenografts. **e.** Body weight corrected for tumor weight from NIH3T3 ETV6-NTRK3 allografted mice as function of time and of altiratinib (15 mg/kg) versus vehicle treatment. BID – bis in die (twice a day). PO – per os (oral treatment). **f.** Body weight corrected for tumor weight from KM-12 xenografted mice as function of time and of altiratinib (3.75, 7.5, and15 mg/kg) versus vehicle treatment. BID, PO.

Supplementary Figure 3

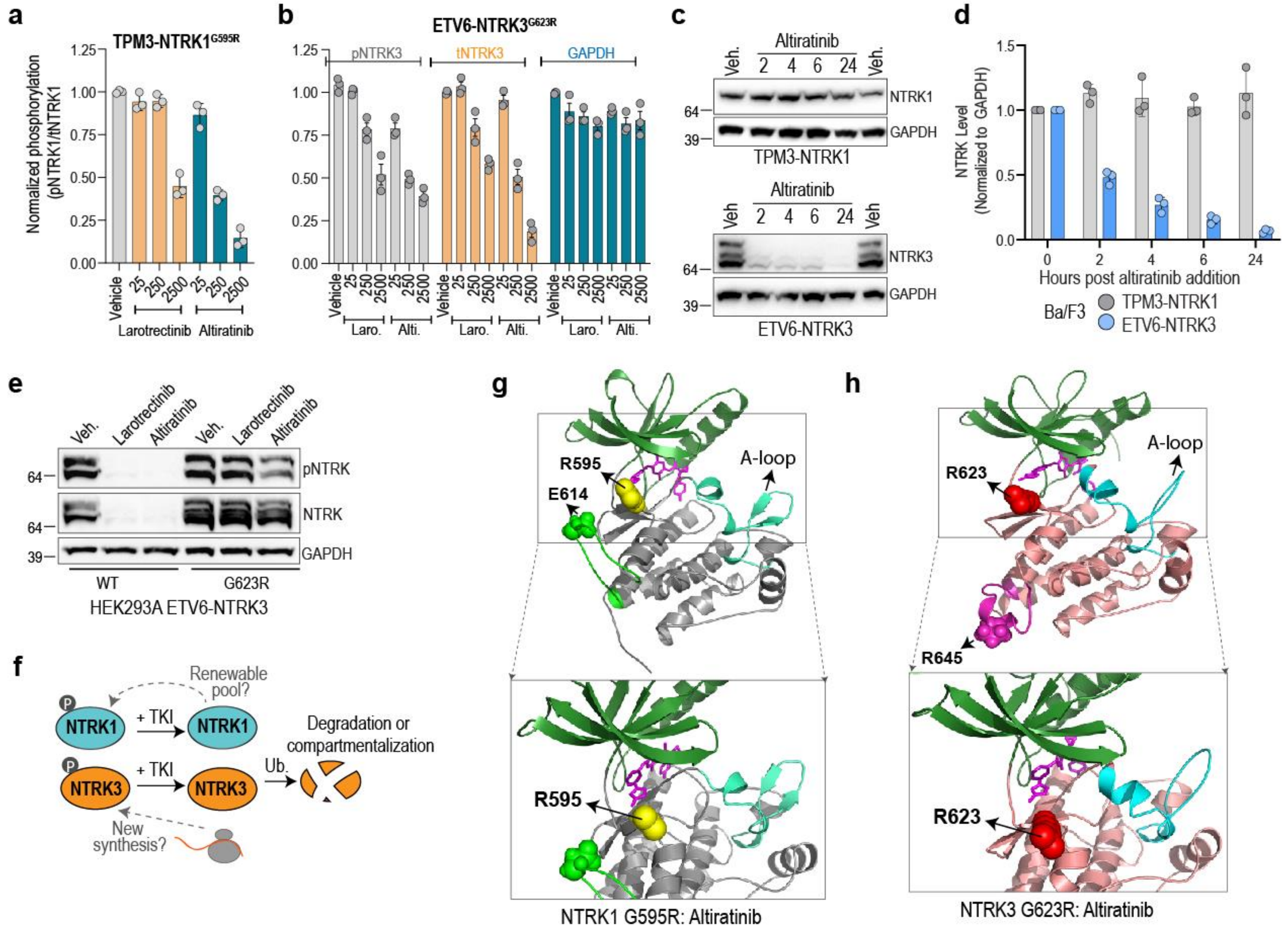

Supplementary Figure 3. Downregulation of ETV6-NTRK3 but not TPM3-NTRK1 after catalytic inhibition and structural differences between NTRK^{G595R} and NTRK1^{G623R}. a. Densitometry data of normalized TPM3-NTRK1^{G595R} phosphorylation (phospho-NTRK1/total NTRK1) after larotrectinib or altiratinib treated Ba/F3 cells expressing the mutant NTRK1 fusion (see Figure 2D for original immunoblot data). Average ± standard error of means (SEM) from three experiments is shown. **b.** Densitometry of phospho-NTRK3 (pNTRK3), total NTRK3 (tNTRK3), and GAPDH levels (loading control) from immunoblots with Ba/F3 ETV6-NTRK3^{G623R} lysates prepared from cells treated with indicated inhibitor concentrations. Average ± standard error of means (SEM) from three experiments is depicted in bar graphs. **c.** TPM3-NTRK1 (top) and ETV6-NTRK3 (bottom) and GAPDH assessed with immunoblotting of Ba/F3 cell lysates after treatment with 25 nM altiratinib for 2, 4, 6, and 24 hours as compared with vehicle (Veh. (0.01% DMSO)) treatment. **d.** Densitometry of TPM3-NTRK1 and ETV6-NTRK3 levels normalized to GAPDH from three experiments. Average ± standard error of means is shown. Asterisk indicates statistical difference in level of ETV6-NTRK2 in 2, 4, 6 and 24 hour altiratinib treated samples compared to vehicle ($p < 0.05$, student t-test). **e.** Immunoblotting for phospho-NTRK (pNTRK), total NTRK (tNTRK) and GAPDH from HEK293A cell lysates transiently transfected with ETV6-NTRK3 wildtype (WT) and ETV6-NTRK3^{G623R} and treated with 75 nM larotrectinib and altiratinib for 2 hours. **f.** Hypothetical model for ETV6-NTRK3 downregulation in case of catalytic inactivity. **g** & **h**. Structural models of NTRK1^{G595R} and NTRK3^{G623R} docked with altiratinib.

Supplementary Figure 4

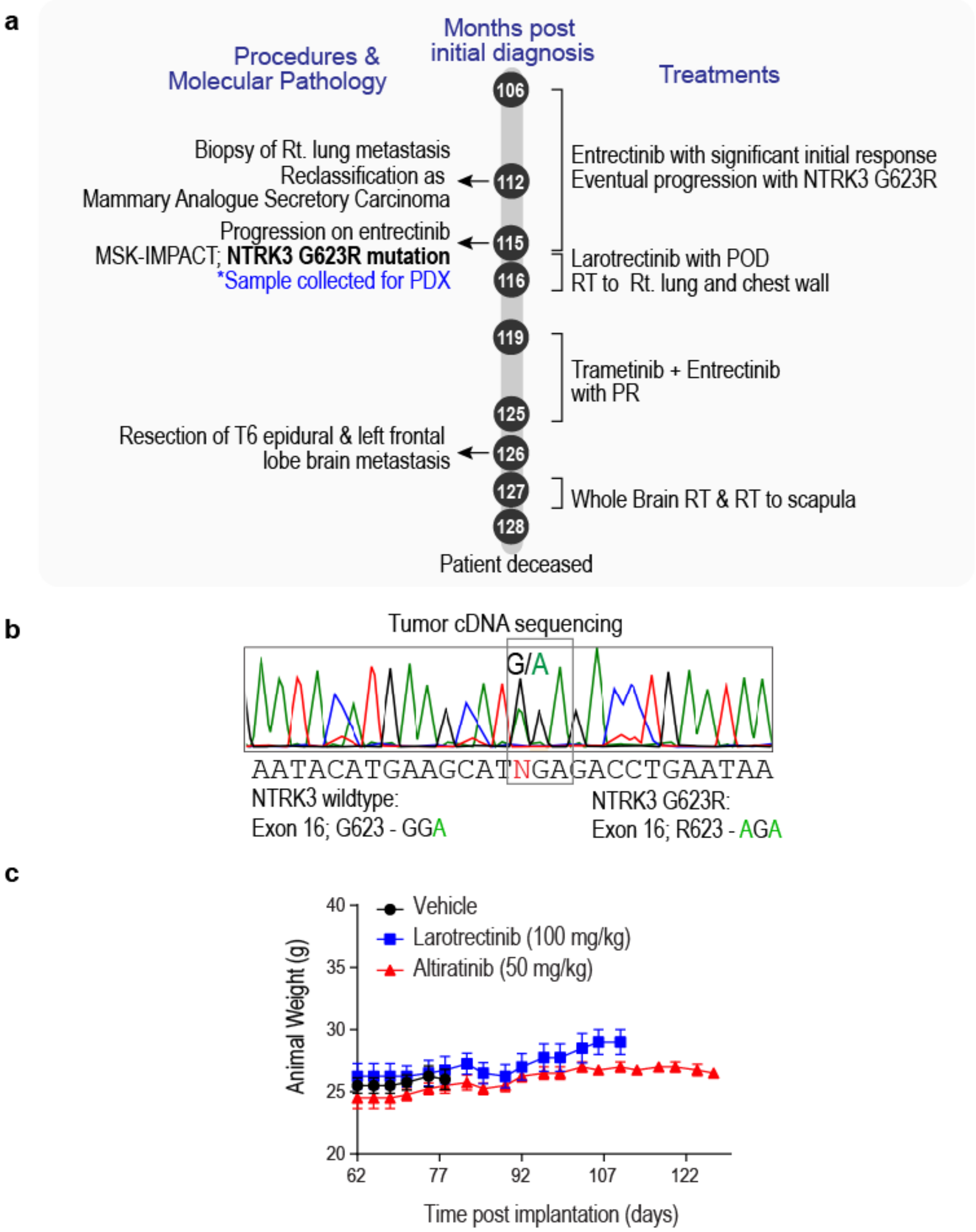

Supplementary Figure 4. Characterization of MASC-0001 PDX. a. Clinical course of patient from whose tumor MASC-0001 PDX was generated. **b.** Sanger sequencing of tumor cDNA shows subclonal NTRK3^{G623R} mutations. **c.** Animal weight measured during treatment with vehicle, larotrectinib (100 mg/kg, BID), and altiratinib (50 mg/kg, BID).

Supplementary Figure 5

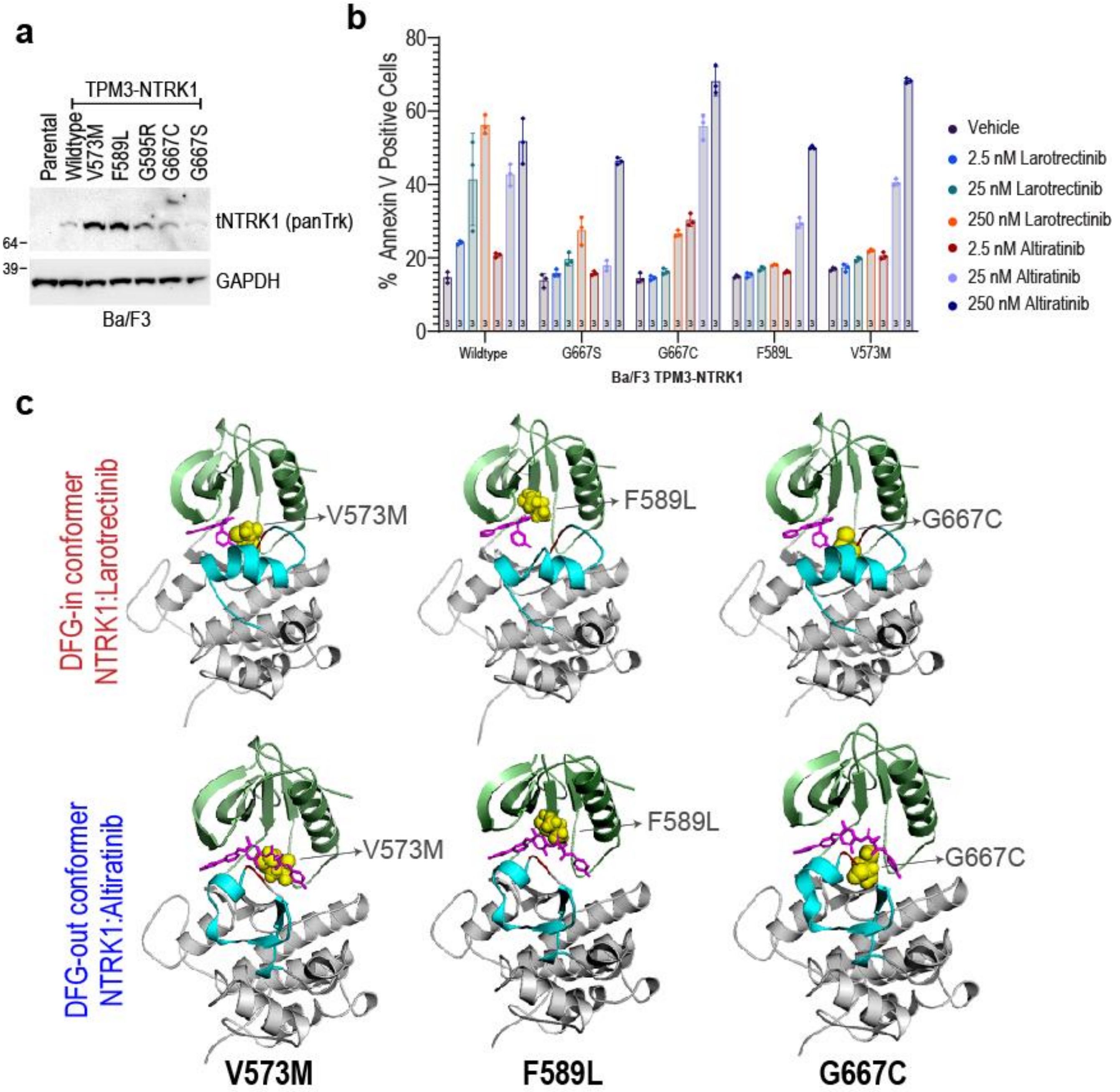

Supplementary Figure 5. Expression of TPM3-NTRK1 variants, apoptosis evaluation and structural modeling of NTRK1 mutants. a. Immunoblot analysis of total TPM3-NTRK1 (tNTRK1, panTrk) and loading control (GAPDH) levels from stable Ba/F3 cells lines engineered with retroviral particles. **b.** Annexin V positive staining of Ba/F3 cell lines treated for 24 hours with larotrectinib and altiratinib at 2.5, 25 and 250 nM, measured using Guava table top flow cytometer. Graph depicts average ± standard error of means and individual values from three replicate wells are shown as icons at the top of each column. **c.** Molecular models of NTRK1^{V573M}, NTRK1^{F589L} and NTRK1^{G667C} docked with larotrectinib or altiratinib in their DFG-in or DFG-out conformations depicted as ribbon diagrams of the kinase domain.

Supplementary Figure 6

a

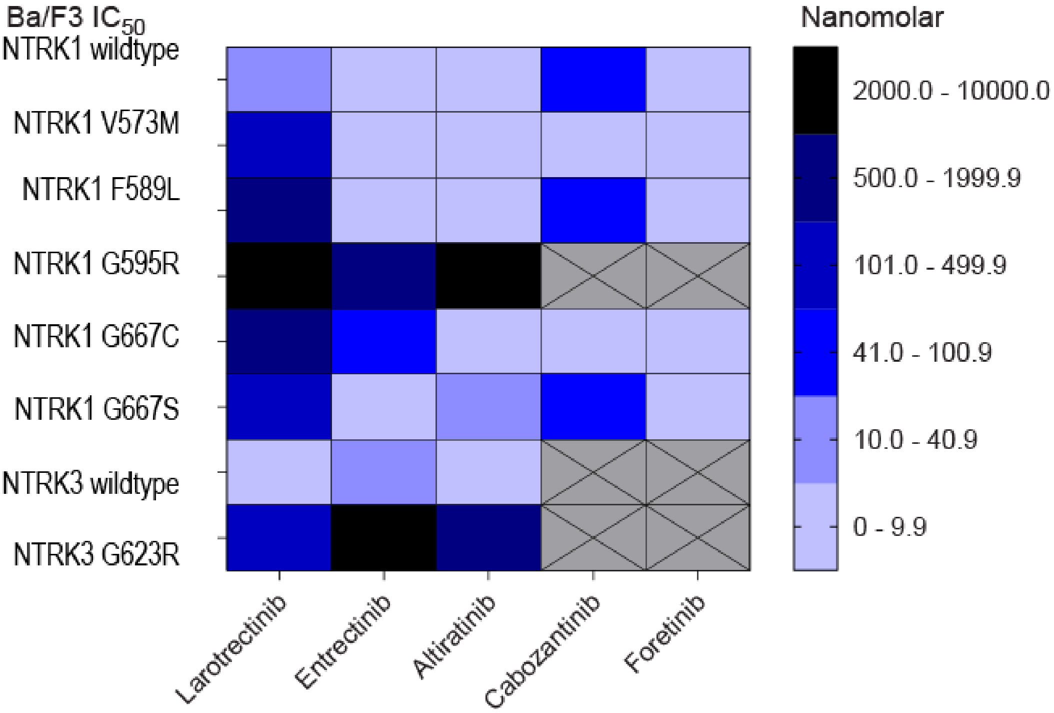

b

Cell Line	Larotrectinib	Entrectinib	Altiratinib	Cabozantinib	Foretinib
NTRK1 wildtype	26.4	2.63	4.75	73.05	8.36
NTRK1 V573M	438.3	6.85	2.34	9.62	2.78
NTRK1 F589L	1800.0	3.49	6.32	71.44	7.16
NTRK1 G595R	>2000	808.1	>2000	Not tested	Not tested
NTRK1 G667C	976.0	71.75	1.69	4.35	0.88
NTRK1 G667S	396.0	8.69	22.39	54.37	2.65
NTRK3 wildtype	7.8	17.2	3.4	Not tested	Not tested
NTRK3 G623R	>2000	882.3	240.0	Not tested	Not tested

Table lists cell-based IC_{50} values in nanomolar (nM) units

Supplementary Figure 6. Heatmap and table summary of type I and type II NTRK inhibitor IC_{50s} determined using Ba/F3 cell model. a. Heatmap with corresponding legend shows relative sensitivity and resistance of the various mutations for larotrectinib, entrectinib, altiratinib, cabozantinib and foretinib. **b.** Table lists specific IC_{50} values resulting cell-based viability assays.

Supplementary Figure 7

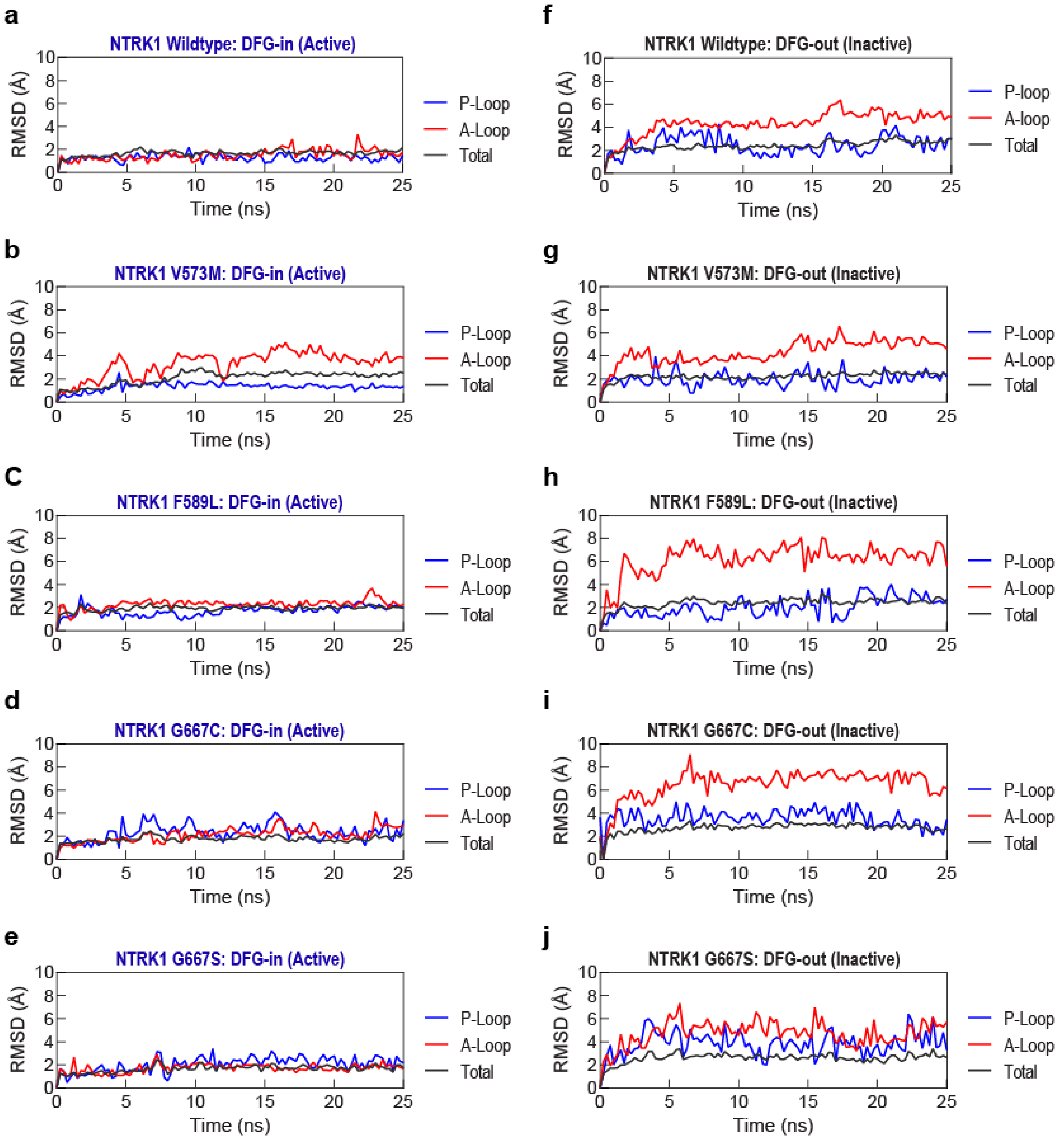

Supplementary Figure 7. NTRK1 (TrkA) kinase domain kinetics. Root Mean Square Deviation (RMSD) of NTRK1 wildtype and mutant kinases during molecular dynamic simulation is shown, as indicated by labels, with the DFG-in (active) conformations in panels **a-e**, and the DFG-out (inactive) conformations in panels **f-j**.

Supplementary Table 1.

Sequences of primers used for RT-PCR

**Sequences of primers**		**Conditions**
	**Sequences 5' to 3'**	
ETV6 Ex 5 Fwd	CCACATCATGGTCTCTGTCTCCC	58.2, 30 sec, 36 cycles
NTRK3 Ex 15 Rev	CTCGCTTCAGCACGATGTCTCT	
GAPDH Fwd	GTGTTTATATGCACATTGGGAG	60, 30 sec, 36 cycles
GAPDH Rev	GGCGCTGAGTACGTCGTGGAGTCCA	
