# Supplementary material for: NTRK kinase domain mutations in cancer variably impact sensitivity to type I and type II inhibitors
Source: Commun Biol. 2020 Dec 16;3:776. doi: 10.1038/s42003-020-01508-w (PMC7745027; doi:10.1038/s42003-020-01508-w)
Supplement: Supplementary file 2 — Reporting Summary [file 42003_2020_1508_MOESM2_ESM.pdf]

nature research

Corresponding author(s): Monika A. Davare

Last updated by author(s): 2020.10.26

Reporting Summary

Nature Research wishes to improve the reproducibility of the work that we publish. This form provides structure for consistency and transparency in reporting. For further information on Nature Research policies, see Authors & Referees and the Editorial Policy Checklist .

Statistics

For all statistical analyses, confirm that the following items are present in the figure legend, table legend, main text, or Methods section.

n/a	Confirmed	
☐	☒	The exact sample size (*n*) for each experimental group/condition, given as a discrete number and unit of measurement
☐	☒	A statement on whether measurements were taken from distinct samples or whether the same sample was measured repeatedly
☐	☒	The statistical test(s) used AND whether they are one- or two-sided<br>*Only common tests should be described solely by name; describe more complex techniques in the Methods section.*
☐	☐	A description of all covariates tested
☐	☐	A description of any assumptions or corrections, such as tests of normality and adjustment for multiple comparisons
☐	☐	A full description of the statistical parameters including central tendency (e.g. means) or other basic estimates (e.g. regression coefficient) AND variation (e.g. standard deviation) or associated estimates of uncertainty (e.g. confidence intervals)
☐	☒	For null hypothesis testing, the test statistic (e.g. *F*, *t*, *r*) with confidence intervals, effect sizes, degrees of freedom and *P* value noted<br>*Give P values as exact values whenever suitable.*
☐	☐	For Bayesian analysis, information on the choice of priors and Markov chain Monte Carlo settings
☐	☐	For hierarchical and complex designs, identification of the appropriate level for tests and full reporting of outcomes
☐	☐	Estimates of effect sizes (e.g. Cohen's *d*, Pearson's *r*), indicating how they were calculated

Our web collection on statistics for biologists contains articles on many of the points above.

Software and code

Policy information about availability of computer code

Data collection	Homology modeling and inhibitor docking studies were conducted using the YASARA software, and homology models were verified using PROCHECK module of PDBSum server.
Data analysis	The following commercially available software was used for data analysis and representation: Microsoft Excel, GraphPad Prism, ImageLab Software (BIO-RAD), Adobe Photoshop and Adobe Illustrator

For manuscripts utilizing custom algorithms or software that are central to the research but not yet described in published literature, software must be made available to editors/reviewers. We strongly encourage code deposition in a community repository (e.g. GitHub). See the Nature Research guidelines for submitting code & software for further information.

Data

Policy information about availability of data

All manuscripts must include a data availability statement. This statement should provide the following information, where applicable:

- Accession codes, unique identifiers, or web links for publicly available datasets
- A list of figures that have associated raw data
- A description of any restrictions on data availability

The datasets generated during and/or analysed during the current study are available from the corresponding author on reasonable request.

Field-specific reporting

Please select the one below that is the best fit for your research. If you are not sure, read the appropriate sections before making your selection.

☒ Life sciences ☐ Behavioural & social sciences ☐ Ecological, evolutionary & environmental sciences

For a reference copy of the document with all sections, see nature.com/documents/nr-reporting-summary-flat.pdf

Life sciences study design

All studies must disclose on these points even when the disclosure is negative.

Sample size	For animal studies, 5 mice per group was planned as this design has allowed us to reliably detect differences of 25% or more between groups over the many years that we have performed animal work. In one instance we had 4 mice per group as not all tumors grew and an ethical decision was made to proceed with the smaller sample size instead of sacrificing the mice and starting all over again. For in vitro viability studies there were at least 3 replicates of each condition in three to five independent experiments, unless in case of rare exception due to technical or COVID-19 related experimental bandwidth reasons as noted.
Data exclusions	No data was excluded from any analysis.
Replication	All in vitro dose-response cell viability experiments included a minimum of three independent replicates in each experiment, and the experiment was repeated at least three and up to five times. All data were highly consistent, and either one representative experiment or an average of all independent experiments was chosen for illustration in the figures.
Randomization	For animal studies tumor bearing mice were assigned to groups so the average tumor volume of each group at start of treatment was similar.
Blinding	No blinding was used in data collection or analysis. All data collected were in specific categories and were quantitative. No data was collected and analyzed by the same person.

Reporting for specific materials, systems and methods

We require information from authors about some types of materials, experimental systems and methods used in many studies. Here, indicate whether each material, system or method listed is relevant to your study. If you are not sure if a list item applies to your research, read the appropriate section before selecting a response.

Materials & experimental systems

n/a	Involved in the study	
☐	☒	Antibodies
☐	☒	Eukaryotic cell lines
☐	☐	Palaeontology
☐	☒	Animals and other organisms
☐	☐	Human research participants
☐	☒	Clinical data

Methods

n/a	Involved in the study	
☐	☐	ChIP-seq
☐	☒	Flow cytometry
☐	☐	MRI-based neuroimaging

Antibodies

Antibodies used	All antibodies used for Western blotting were obtained from either Cell Signaling Technology or In vitrogen and have been used extensively in published literature. Antibodies used for IHC were obtained from Abcam, Dako or Leica Biosystems, and these antibodies have been validated and routinely used for clinical diagnosis at MSKCC and many other institutions. The catalog number of each antibody and specificity is listed in Supplementary Methods.
Validation	Positive controls that are used on clinical diagnosis were included on IHC studies. All Western blotting antibodies were validated by the commercial provider and data confirmed in many publications by independent research groups over many years.

Eukaryotic cell lines

Policy information about cell lines

Cell line source(s)	Original Ba/F3 cells were procured from DSMZ (Catalog # ACC 300). The cells have existed in the laboratory for some time now. HEK293A cells were used for transient transfection. These were originally procured from Thermo Scientific (Catalog # R70507), and have been sub-cultivated in the laboratory for several years now. Platinum-E (Plat-E) Retroviral Packaging Cell Line was purchased from Cell BioLabs, Inc.
Authentication	Ba/F3 cells are exquisitely dependent on the cytokine IL-3. All experiments involve the requisite control of IL-3 withdrawal to ensure reproducible Ba/F3 cell death in 96 hours. HEK293A and Platinum-E cells were not authenticated as they were only used transiently for expressing protein or generating retrovirus.
Mycoplasma contamination	Cell lines were tested for mycoplasma once every 3 months. One incidence of mycoplasma contamination occurred with four Ba/F3 stable cell lines. At this point, these cells were treated for anti-mycoplasma antibiotics for three consecutive weeks, and retested after antibiotic washout for one week. The cells were cleared and responded to inhibitors no differently after treatment with anti-mycobacterial reagent.

Commonly misidentified lines (See ICLAC register)	None.

Palaeontology

Specimen provenance	Not relevant to this study
Specimen deposition	Not relevant to this study
Dating methods	Not relevant to this study

☐ Tick this box to confirm that the raw and calibrated dates are available in the paper or in Supplementary Information.

Animals and other organisms

Policy information about studies involving animals; ARRIVE guidelines recommended for reporting animal research

Laboratory animals	All studies were conducted in Nod Scid gamma (NSG) strain mice. All animals were female and used at 6 weeks of age.
Wild animals	This study did no involve wild animals.
Field-collected samples	This study does not include samples collected in the field.
Ethics oversight	All mice were cared for in accordance with guidelines approved by the Memorial Sloan Kettering Cancer Center Institutional Animal Care and Use Committee and Research Animal Resource Center and animals were monitored daily.

Note that full information on the approval of the study protocol must also be provided in the manuscript.

Human research participants

Policy information about studies involving human research participants

Population characteristics	No human subjects were involved in this study
Recruitment	No human subjects were involved in this study
Ethics oversight	No human subjects were involved in this study

Note that full information on the approval of the study protocol must also be provided in the manuscript.

Clinical data

Policy information about clinical studies

All manuscripts should comply with the ICMJEguidelines for publication of clinical research and a completedCONSORT checklist must be included with all submissions.

Clinical trial registration	Not relevant to this study
Study protocol	Not relevant to this study
Data collection	Not relevant to this study
Outcomes	Not relevant to this study

ChIP-seq

Data deposition

☐ Confirm that both raw and final processed data have been deposited in a public database such as GEO.

☐ Confirm that you have deposited or provided access to graph files (e.g. BED files) for the called peaks.

Data access links *May remain private before publication.*	Not relevant to this study
Files in database submission	Not relevant to this study
Genome browser session (e.g. UCSC)	Not relevant to this study

Methodology

Replicates	Not relevant to this study
Sequencing depth	Not relevant to this study
Antibodies	Not relevant to this study
Peak calling parameters	Not relevant to this study
Data quality	Not relevant to this study
Software	Not relevant to this study

Flow Cytometry

Plots

Confirm that:

- ☐ The axis labels state the marker and fluorochrome used (e.g. CD4-FITC).
- ☐ The axis scales are clearly visible. Include numbers along axes only for bottom left plot of group (a 'group' is an analysis of identical markers).
- ☐ All plots are contour plots with outliers or pseudocolor plots.
- ☐ A numerical value for number of cells or percentage (with statistics) is provided.

Methodology

Sample preparation	Flow cytometry was only used to enrich the NTRK-fusion expressing population before IL-3 withdrawal to make a stable cell line. The retroviral vector contains mCherry and this served as a surrogate marker for retroviral integration. Other than use flow cytometry to enrich the mCherry expressing cells, we did not perform any quantitative flow cytometry, and do not think the questions are highly relevant to this use case. Particularly because the more rigorous step in creating a stable Ba/F3 NTRK-fusion cell line is the IL-3 withdrawal step, where any cell that has sub-threshold or no NTRK-fusion expression underwent cell death and the remaining surviving cell population (minus IL-3) was considered the stable cell line.
Instrument	*Identify the instrument used for data collection, specifying make and model number.*
Software	*Describe the software used to collect and analyze the flow cytometry data. For custom code that has been deposited into a community repository, provide accession details.*
Cell population abundance	*Describe the abundance of the relevant cell populations within post-sort fractions, providing details on the purity of the samples and how it was determined.*
Gating strategy	*Describe the gating strategy used for all relevant experiments, specifying the preliminary FSC/SSC gates of the starting cell population, indicating where boundaries between "positive" and "negative" staining cell populations are defined.*

☐ Tick this box to confirm that a figure exemplifying the gating strategy is provided in the Supplementary Information.

Magnetic resonance imaging

Experimental design

Design type	Not relevant to this study
Design specifications	Not relevant to this study
Behavioral performance measures	Not relevant to this study

Acquisition

Imaging type(s)	Not relevant to this study
Field strength	Not relevant to this study
Sequence & imaging parameters	Not relevant to this study
Area of acquisition	Not relevant to this study

Diffusion MRI ☐ Used ☐ Not used

Preprocessing

Preprocessing software	Not relevant to this study
Normalization	Not relevant to this study
Normalization template	Not relevant to this study
Noise and artifact removal	Not relevant to this study
Volume censoring	Not relevant to this study

Statistical modeling & inference

Model type and settings	Not relevant to this study
Effect(s) tested	Not relevant to this study

Specify type of analysis: ☐ Whole brain ☐ ROI-based ☐ Both

Statistic type for inference (See Eklund et al. 2016)	Not relevant to this study
Correction	Not relevant to this study

Models & analysis

n/a	Involved in the study
☐	☐ Functional and/or effective connectivity
☐	☐ Graph analysis
☐	☐ Multivariate modeling or predictive analysis

Functional and/or effective connectivity	Not relevant to this study
Graph analysis	Not relevant to this study
Multivariate modeling and predictive analysis	Not relevant to this study
